# Using Theories, Models, and Frameworks to Inform Implementation Cycles of Computerized Clinical Decision Support Systems in Tertiary Health Care Settings: Scoping Review

**DOI:** 10.2196/45163

**Published:** 2023-10-18

**Authors:** Manasha Fernando, Bridget Abell, Zephanie Tyack, Thomasina Donovan, Steven M McPhail, Sundresan Naicker

**Affiliations:** 1 Australian Centre for Health Services Innovation and Centre for Healthcare Transformation School of Public Health and Social Work, Faculty of Health Queensland University of Technology Brisbane Australia; 2 Digital Health and Informatics Directorate Metro South Health Brisbane Australia

**Keywords:** computerized clinical decision support systems, CDSS, implementation science, hospital, theories, models, frameworks, mobile phone

## Abstract

**Background:**

Computerized clinical decision support systems (CDSSs) are essential components of modern health system service delivery, particularly within acute care settings such as hospitals. Theories, models, and frameworks may assist in facilitating the implementation processes associated with CDSS innovation and its use within these care settings. These processes include context assessments to identify key determinants, implementation plans for adoption, promoting ongoing uptake, adherence, and long-term evaluation. However, there has been no prior review synthesizing the literature regarding the theories, models, and frameworks that have informed the implementation and adoption of CDSSs within hospitals.

**Objective:**

This scoping review aims to identify the theory, model, and framework approaches that have been used to facilitate the implementation and adoption of CDSSs in tertiary health care settings, including hospitals. The rationales reported for selecting these approaches, including the limitations and strengths, are described.

**Methods:**

A total of 5 electronic databases were searched (CINAHL via EBSCOhost, PubMed, Scopus, PsycINFO, and Embase) to identify studies that implemented or adopted a CDSS in a tertiary health care setting using an implementation theory, model, or framework. No date or language limits were applied. A narrative synthesis was conducted using full-text publications and abstracts. Implementation phases were classified according to the “Active Implementation Framework stages”: exploration (feasibility and organizational readiness), installation (organizational preparation), initial implementation (initiating implementation, ie, training), full implementation (sustainment), and nontranslational effectiveness studies.

**Results:**

A total of 81 records (42 full text and 39 abstracts) were included. Full-text studies and abstracts are reported separately. For full-text studies, models (18/42, 43%), followed by determinants frameworks (14/42,33%), were most frequently used to guide adoption and evaluation strategies. Most studies (36/42, 86%) did not list the limitations associated with applying a specific theory, model, or framework.

**Conclusions:**

Models and related quality improvement methods were most frequently used to inform CDSS adoption. Models were not typically combined with each other or with theory to inform full-cycle implementation strategies. The findings highlight a gap in the application of implementation methods including theories, models, and frameworks to facilitate full-cycle implementation strategies for hospital CDSSs.

## Introduction

### Background

Data in tertiary health care settings, including hospitals, are now frequently digitized through the integration of electronic health records and other systems. The embedding of computerized clinical decision support systems (CDSSs) that leverage digital clinical information to support the safe and effective provision of health services has become increasingly widespread, particularly in hospitals [1-3]. There has been growing evidence to support the application of CDSS innovations to streamline clinical management decisions [4], improve risk-based decision-making, and provide personalized care [5] within acute health care settings. This includes computerized provider order entry (CPOE) systems for medical administration [6,7], interruptive alerts to promote patient safety [4], and artificial intelligence for risk prediction or diagnostic decision support [8-10], among other applications. The effective implementation of a CDSS rests upon a multifaceted interplay among various human intermediaries (such as patients, researchers, IT specialists, health care decision makers, and physicians), integrated computerized systems, the CDSS interface, the knowledge embedded within the decision support system, and the broader health care [11]. Although other types of health information technology systems may share similar determinants [12], the interactivity of CDSSs coupled with their role in supporting clinical decision-making, add additional layers of consideration to address potential barriers and facilitators to implementation. Even the implementation of a seemingly “simple” CDSS alert and reminder system necessitates a thoughtful assessment of alert design and clinical workflows as potential barriers to adoption [13].

Despite their widespread adoption in modern health care systems, studies have observed suboptimal use of CDSS innovations over time [14-17]. Furthermore, implementing a CDSS innovation in clinical practice did not ensure changes in prescribing choices, according to a 2010 comprehensive review (n=58 trials) [16]. This review identified that a lack of technical training and a low acceptance of CDSS outputs acted as barriers to end-user adoption of CDSS innovations [16]. A more recent 2022 systematic review and meta-analysis (n=11) on the impact of CDSS on provider behavior in inpatient settings found no statistically significant increase in clinician adoption of the desired practice behavior after CDSS implementation [17]. The authors suggested that contextual determinants such as increased workflow interruptions or “CDSS frustrations” may adversely change provider behavior and CDSS interaction during implementation [17]. The differences in how well CDSS innovations can change clinical behavior and consequently impact clinical practice and patient outcomes might stem from the ineffectiveness of the CDSS innovation on its own, the impact of wider contextual factors after implementation, or interactions between the 2. Several other systematic reviews across varied clinical contexts (ie, inpatient hospital care) have been conducted to measure clinical effectiveness, provider uptake, or change in clinical practice owing to CDSS implementation [18-23]. Overall, these systematic reviews have demonstrated either no significant effect or small effect sizes of CDSS innovations to improve clinical processes and patient outcomes [18-23].

A range of factors have been reported to hinder the adoption of CDSSs, including usability, clinical workflow disruption, constrained financing systems, and medico-legal concerns, among other wider organizational and contextual barriers [14,15,24,25]. The implementation process and the mechanisms for success or failure of CDSS uptake are likely multifactorial; however, the appropriate use of tools and processes to support the identification of these factors and subsequent adoption have typically lacked systematic planning and evaluation [24,26]. Consequently, CDSS innovations and associated implementation strategies may not be contextually tailored to local health system settings [24,27]. This may account for increases in inappropriate use [28], suboptimal uptake [22], and abandonment over time of the CDSS innovation and related implementation strategies [14]. Given that CDSS often include multiple intervention components that need to be contextually adopted in fast-paced tertiary health care settings such as hospitals, mitigating the factors reported to hinder the adoption of CDSS will require evidence-based decision-making and planning [22,23].

Theories, models, and frameworks from the field of implementation science may assist in providing translational guidance for CDSS implementation and promoting its adoption in complex hospital settings. Implementation science can be defined as “the scientific study of methods to promote the systematic uptake of research findings into routine practice to improve the quality and effectiveness of routine services” [29].

Nilsen [30] conducted a review of the types and uses of theory within the field of implementation science, which was used to create a taxonomy of 5 categories of theories, models, and frameworks. Their review led to the creation of a taxonomy comprising 5 categories encompassing theories, models, and frameworks. One category, referred to as “process models,” includes models that outline specific phases, steps, or stages for translating research into practice. An example of this is the 3-phase structure within the knowledge-to-action framework [31]. Another category involves “deterministic frameworks,” which encompass frameworks such as the Consolidated Framework for Implementation Research (CFIR) [32]. These frameworks incorporate descriptive categories that account for the factors influencing various aspects of the process, such as the intervention, adopters, end users, context, and strategy. The third category, “classic theories,” consists of well-established theories originating from fields such as psychology, sociology, and organizational management, for instance, the social cognitive theory [33]. The fourth category is “implementation theories,” which are novel concepts derived from multiple disciplines and are still in the process of empirical testing; an example of such a theory is the theoretical domain’s framework [34].

A separate and distinct component of the implementation methodology is “implementation evaluation.” This involves evaluating the effectiveness of the implementation strategies, and the methodologies designed for this purpose can be theories, models, or frameworks. Notable examples include the Reach, Effectiveness, Adoption, Implementation, and Maintenance framework [35] and the Predisposing, Reinforcing, and Enabling Constructs in Educational Diagnosis and Evaluation–Policy, Regulatory, and Organizational Constructs in Educational and Environmental Development model [36].

Despite the existence of a range of theories, models, and frameworks applied across health care settings to support digital health implementation strategies, they are not commonly used [37] in practice, particularly in hospitals [27,38]. The reasons for this gap are not well known but could be owing to a lack of awareness; uncertainty about using theories, models, or frameworks; or hesitancy to apply them in practice [39]. This is especially noted in the field of digital health, which often necessitates the adoption of multifaceted interventions in fast-paced and often resource-constrained health care settings [27,39]. To date, there has been 1 prior review of theoretical implementation frameworks for CDSSs that examined 16 studies published from 2005 to 2014 [40]. This review identified 15 different theoretical and conceptual frameworks influencing adoption, with the technology acceptance model being the most used framework [40]. However, the review did not focus on hospital (singular) settings, models and frameworks were not differentiated in the findings, and the rationale for using select theoretical approaches was not noted. Consequently, the use and application of a theory, model, or framework were not examined [40].

### Objectives

There has been a proliferation of models and frameworks specific to the adoption of health system interventions in practice, including health information technology [12,41]. However, it is unclear how frequently, if at all, these theories, models, and frameworks have been used to support the implementation of CDSS tools in tertiary health care settings. Furthermore, little is known about their contextual application or efficacy in promoting CDSS uptake. Even when a theory, model, or framework has been applied to a study, theoretical constructs have been found to be chosen inappropriately [42], not adhered to when applied [43-45], and often applied retrospectively after implementation when many approaches are designed to be applied prospectively [46]. As a theoretical or hypothetical understanding can shape implementation efforts, it is important to review not only what theory, model, or framework is being used (if at all) but also how it is being informed and used. This scoping review aims to identify which theories, models, or frameworks have been used to facilitate the prospective implementation and adoption of CDSSs in tertiary health care settings, including hospitals. Furthermore, it will also describe (only if mentioned within papers) any rationales or justifications for selecting a particular theory, model, or framework. This review will also identify the implementation phases for each included full-text paper using the “Active Implementation Framework” [47,48].

## Methods

### Overview

The review was appraised using the PRISMA-ScR (Preferred Reporting Items for Systematic Reviews and Meta-Analyses extension for Scoping Reviews) checklist (Multimedia Appendix 1). The review was guided by the Joanna Briggs Institute Manual for Evidence Synthesis [49] and used the 5 steps from the methodological framework outlined by Arksey and O’ Malley [50]. The following activities were conducted: identifying the research question; identifying the relevant studies; selecting records; charting the data; and collating, summarizing, and reporting the results.

### Identifying Relevant Studies

The search (date last searched: May 2021) was conducted in the following databases: CINAHL via EBSCOhost, PubMed, Scopus, PsycINFO, and Embase using relevant Medical Subject Heading headings and key terms, including “implementation,” “theory,” “model,” “framework,” “computerized clinical decision support system,” and “hospital.” The final search strategy was refined using the Institute for Evidence-Based Healthcare Systematic Review Accelerator Search Refinery [51] to ensure that relevant studies were being captured. The search terms used for each database can be found in Multimedia Appendix 2.

### Eligibility Criteria

Records were included if they met all the eligibility criteria outlined in Textbox 1. No date or language limits were applied to exhaustively capture CDSS implementation studies. Abstracts were included in the review to capture the rapid pace of knowledge generation in the fields of digital health and implementation [52,53]. A CDSS was defined as software intended to be a direct aid to clinical decision-making. In this system, patient characteristics are matched to a computerized clinical knowledge base with specific assessments or recommendations, which are available to the clinician for a decision at the point of care. Examples of CDSSs include web applications, CPOE systems, desktops, smartphones, tablets, and devices for biometric monitoring or wearable health technology, which can be linked to electronic health record databases [1]. The inclusion criteria were restricted to prospective studies, as studies describing retrospective activities involving the mapping of theory, models, or frameworks were considered nonactive implementation activities, falling outside the purview of this review. Furthermore, retrospective assessments do not entail real-time analyses of deliberate implementation strategies in action. Instead, retrospective studies may involve evaluations facilitated by theories, models, or frameworks to distinguish effective from ineffective implementation or intervention adoption processes that have already occurred [30]. As a result, such studies do not lend themselves to exploring the advantages of using theories, models, or frameworks for implementation [46]. Rather, they contribute to understanding the mechanics of successful implementation and its outcomes.

The perspectives adopted for this review were those of end users within the health system, such as clinicians or researchers. Consequently, studies that focused on patient perspectives and feedback stemming from information retrieved during clinical management were excluded from this scoping review [16,54]. Typically, CDSSs forms part of tertiary health care infrastructure that is generally unseen by patients themselves. Consequently, decisions to adopt, expand, or use these tools are made by health service leaders, often without consumer consultation [16,54]. The categories of the taxonomy by Nilsen [30] were used to classify the approaches reported as either models, determinant frameworks, evaluation frameworks, or theories. Further operational definitions can be found in Multimedia Appendix 3.

**Inclusion and exclusion criteria.**

**Inclusion criteria**

- Examined implementation of a computerized clinical decision support system (CDSS) into clinical or routine practice
- CDSS designed for use at the point of care
- Prospective implementation of CDSS
- Conducted in a hospital or tertiary health care setting
- Reported using a theory, model, or framework in relation to the implementation of a CDSS (the definitions and elaboration of terms used to identify theories, models, or frameworks according to the taxonomy by Nilsen [30] are provided in Multimedia Appendix 3 [1,30,54-57])

**Exclusion criteria**

- Conducted solely in a primary care or community setting such as general practice or allied health care clinics
- Developed a theory, model, or framework but did not apply it to a CDSS implementation
- Described the development of a CDSS
- Retrospectively evaluated a CDSS
- Studied patient decision-making as opposed to clinical decision-making

### Study Selection

Search results from each database were imported into EndNote X9 (Clarivate), and duplicates were removed. The remaining records were then uploaded to Rayyan Beta (Rayyan Systems Inc) [58] for additional deduplication and screening. A title and abstract screen of 10% (687/6874) of the included records was conducted in Rayyan Beta by reviewers (MF, BA, and ZT) using the eligibility criteria and the definitions and elaboration document. Following this, the research team met to discuss discrepancies and make modifications to the eligibility criteria and the definitions and elaboration document. When ≥75% agreement was achieved, 2 reviewers (MF and TD) screened the remaining titles and abstracts. Conflicts were resolved by discussion or by an additional reviewer. Full-text publications were then sought for the remaining records.

Full-text records were screened in duplicate by a reviewer pair, with 1 reviewer (MF) reviewing all full texts paired with additional reviewers (TD, BA, ZT, and SN) who each reviewed a portion of the full texts. Conflicts were resolved by discussion or by reviewers BA or SN.

### Charting the Data

The fields for data extraction were adapted from the Joanna Briggs Institute template found in the JBI Manual for Evidence Synthesis [49]. Extraction included the country of origin (where the study was conducted); year of publication; data type (quantitative, qualitative, or mixed); study design; types of CDSSs implemented; clinical context of implementation; implementation phase of the CDSS (as operationally defined by the Active Implementation Framework [47,48]); and the theory, model, or framework used.

The data extraction form was piloted and refined by reviewers SN and MF on 10% (5/42) of the included studies to ensure consistency and clarity in the data extracted. Following this, 1 reviewer (MF) independently extracted data for the 42 included records.

### Collating, Summarizing, and Reporting the Results

The results were synthesized and reported by conducting a narrative synthesis of the extracted data from all included full-text publications and using graphical displays of the extracted information. Using the taxonomy by Nilsen [30], where described, implementation approaches were synthesized into models, determinant frameworks, evaluation frameworks, and theories. If the study used >1 theory, model, or framework, it was noted as a “mixed” approach and described appropriately, accounting for the approaches taken. Where included, rationales, strengths, and limitations of the approach were collated and summarized.

Implementation phases were categorized according to the Active Implementation Framework [47,48] using the following operational definitions: exploration (feasibility and organizational readiness), installation (organizational preparation), initial implementation (initiating implementation, ie, training), and full implementation (sustainment). This framework is a pragmatic categorization tool to identify the phases of digital health implementation using consistent, jargon-free language.

It is important to highlight that studies classified as “other” within the scope of this review were those that met the specified eligibility criteria but did not directly use a theory, model, or framework in the active implementation processes of translating a CDSS into a hospital setting. However, these studies may have used a theory, model, or framework to guide implementation strategies as a component of hybrid implementation-effectiveness trials or analogous experimental methodologies. This classification was applied to maintain a clear distinction between studies directly engaged in CDSS implementation within a hospital context and those adopting a theory, model, or framework to shape implementation strategies within hybrid trials or related experimental approaches in the same setting.

Figures were produced using R Statistical Software (v4.2.1.2; R Foundation for Statistical Computing) [59] and Wickham’s R package *ggplot2* [60]. Results from the included abstracts were summarized separately using narrative synthesis.

## Results

### Overview

The initial searches returned 3411 unique records after duplicate removal, of which 3228 (94.64%) were excluded after title and abstract screening (Multimedia Appendix 4). The full texts of 183 records were sought. The full text of 1 record could not be obtained despite contacting the author and was therefore included as an abstract. Of the 183 records, 44 (24%) met the inclusion criteria and were included in the review (21 abstracts and 23 full-text records).

**Figure 1:**
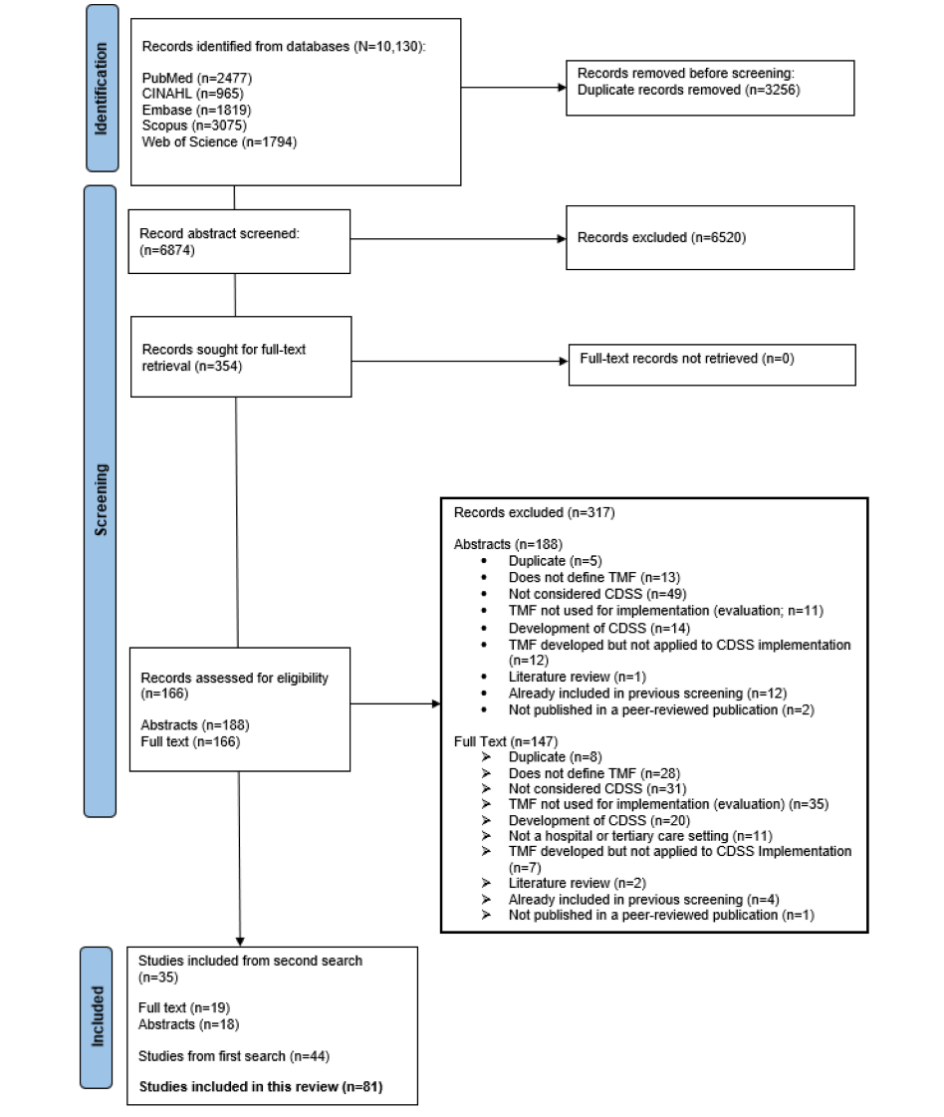
PRISMA (Preferred Reporting Items for Systematic Reviews and Meta-Analyses) flow diagram showing the review process of the updated search [61]. CDSS: computerized clinical decision support system; TMF: theories, models, and frameworks.

An updated search returned 6874 unique records after duplicate removal, of which 6520 (94.85%) were excluded after title and abstract screening, as shown in Figure 1 [61]. The full texts of 354 records were sought. Of the 354 records, 19 (5.4%) full-text records and 18 (5.1%) abstracts from the updated search met the inclusion criteria and were included in this review. Thus, 81 records (39 abstracts and 42 full-text records) were included in this review, as shown in Figure 1.

### Characteristics of Full-Text Published Studies

Publication occurred in a wide range of academic journals, including clinical or discipline-specific journals and informatics or computer science–specific journals. The most frequent country of origin for the full-text studies in this review was the United States (18/42, 43%). The study designs varied considerably (Table 1); the most frequent designs were pre-post designs (15/42, 36%) and case studies or case series (11/42, 26%). There were 3 lower-middle–income countries, as defined by the World Bank [62] (Tunisia [63,64], Nepal [65], and Tanzania [66]) represented in the 42 included full-text studies.

**Table 1:** Characteristics of the included full-text published studies (n=42).

Taxonomy by Nilsen [30], study, and year of publication			Country^a^		Data type		Study design
**Models**							
	Ashraf et al [67], 2022	United States		Mixed		Pre-post	
	Ali et al [68], 2020	Canada		Quantitative		Pre-post	
	Bernonille et al [69], 2011	France, Denmark, and Greece		Mixed		Case series	
	Bertoni et al [70], 2019	United States		Quantitative		Pre-post	
	Cho et al [71], 2023	Korea		Quantitative		Pre-post	
	Ellouzi et al [64], 2017	Tunisia		Quantitative		Case study	
	Flaherty et al [72], 2021	United States		Quantitative		Pre-post	
	Hanna et al [73], 2020	United Kingdom		Mixed		Pre-post	
	Jemal et al [63], 2019	Tunisia		Quantitative		Case study	
	Khazaei et al [74], 2015	Canada		Quantitative		Case series	
	Klunk et al [75], 2021	United States		Mixed		Pre-post	
	Leibowitz et al [76], 2018	United States		Quantitative		Pre-post	
	Maia and Lapão [77], 2017	Portugal		Mixed		Case series	
	Simões et al [78], 2018	Portugal		Mixed		Case series	
	Sinvani et al [79], 2020	United States		Mixed		Case series	
	Stanger et al [80], 2018	United Kingdom		Mixed		Pre-post	
	Streiff et al [81], 2012	United States		Quantitative		Case study	
	Tan et al [82], 2021	Singapore		Mixed		Pre-post	
**Determinant frameworks**							
	Bomba and Land [83], 2006	Australia		Qualitative		Case study	
	Brunner et al [84], 2020	United States		Mixed		Pre-post	
	Chinman et al [85], 2019	United States		Mixed		Stepped-wedge cluster randomized design	
	Förberg et al [86], 2016	Sweden		Quantitative		Cluster randomized trial	
	Geva et al [87], 2021	United States		Mixed		Observational study	
	Kuo and Chang [88], 2014	Taiwan		Quantitative		Pre-post	
	Kawamoto et al [89], 2021	United States		Mixed		Case study	
	Mosch et al [90], 2022	Germany		Qualitative		Qualitative exploratory study	
	Patapovas et al [91], 2013	Germany		Quantitative		Prospective 3-phase study	
	Sheehan et al [92], 2013	United States		Qualitative		Multisite cross-sectional study	
	Shelley et al [93], 2018	United States		Mixed		Cluster randomized controlled trial	
	Stablein et al [94], 2003	United States		Mixed		Cross-sectional Study	
	van Engen-Verheul et al [95], 2016	Netherlands and Ireland		Mixed		Pre-post	
	Wilk et al [96], 2013	United Kingdom		Mixed		Prospective cohort study	
**Evaluation framework**							
	Fuller et al [97], 2020	United States		Mixed		Cross-sectional study	
	Melnick et al [98], 2022	United States		Quantitative		Pragmatic cluster randomized controlled trial	
**Theory**							
	Blandford et al [99], 2022	United Kingdom		Qualitative		Qualitative exploratory study	
**Model and determinant framework**							
	Afshar et al [100], 2023	United States		Mixed		Pre-post	
**Model and evaluation framework**							
	Jacobsohn et al [101], 2022	United States		Mixed		Pre-post	
	Mehanni et al [65], 2019	Nepal		Mixed		Case study	
**Evaluation framework and determinant framework**							
	Paulsen et al [102], 2021	Norway		Qualitative		Qualitative exploratory study	
	Vasudevan et al [66], 2020	Tanzania		Mixed		Effectiveness-implementation hybrid study	
**Theory and determinant framework**							
	Fujimori et al [103], 2022	Japan		Mixed		Cross-sectional study	
	Hsiao and Chen [104], 2016	Taiwan		Quantitative		Cross-sectional study	

^a^Country where the study was conducted.

Out of the 42 full-text studies, 25 (60%) used multiple methods of data collection (ie, quantitative and qualitative). Quantitative data collection *solely* occurred in 31% (13/42) of full-text records, with solely collected qualitative data being less common (4/42, 10%).

### CDSS Characteristics in Full-Text Published Studies

A total of 45 separate CDSSs were reported in the 42 articles included as shown in Table 2. The most commonly reported CDSSs were electronic prescribing or order sets (12/42, 29%) [65,69,70,75,76,79-81,83,88,94,98], followed by checklists, forms, or clinical guidelines (11/42, 26%) [63,66,73,82,89,92,93,95,102,104] and electronic alerts or reminders (9/42, 21%) [67,68,77,78,84,86,91,100,101]. Commonly, CDSSs were implemented in pharmacy or prescription services (17/42, 41%).

Studies that used theory, model, and framework-informed approaches for the planned implementation of CDSS within a single implementation phase described the following: exploration (3/42, 7%), installation (9/42, 21%), initial implementation (4/42, 10%), and full implementation (2/42, 5%). The remaining studies described ≥2 phases: exploration and installation (3/42, 7%); exploration, installation, and initial implementation (4/42, 10%); installation and initial implementation (3/42, 7%); initial implementation and full implementation (1/42, 2%); installation, initial implementation, and full implementation (3 phases; 1/42, 2%); and conducting implementation activities across all 4 phases (3/42, 7%). Another 21% (9/42) of the studies were experimental rather than evaluating or conducting implementation activities directly. These hybrid effectiveness-implementation studies were considered pretranslational studies and classified as “other” [47,48].

The rationale for implementing a CDSS was described in 35 (83%) of the 45 CDSS studies. Rationales included removing the burden from clinicians in complex situations (eg, monitoring antibiotic use [77]). Other rationales cited for implementing a CDSS included the following: the CDSS as a separate intervention to address a clinical need or context (eg, reducing the incidence of hospital-acquired infections [64]), increasing workflow efficacy [79], improving adherence to clinical guidelines [82], transforming time-consuming paper versions of a CDSS to a digital version [81,87,104], engaging or collaborating with health care workers [78], and decreasing duplication of tests or health resource waste (eg, duplicates of magnetic resonance imaging scans) [84]. Other authors noted that their study was a scale-up or modification of an initial pilot of a CDSS [80,82,98], that there was existing evidence that validated the use of a CDSS [66], and contextual factors of the health system (eg, rapid turnover of clinicians) required the implementation of a CDSS [70].

**Table 2:** Characteristics of the computerized clinical decision support systems (CDSSs) implementations included in the full-text studies (n=42).

Taxonomy by Nilsen [30], study, and year of publication			Clinical context		CDSS type		Implementation phase		Theory, model, or framework
**Model**									
	Ashraf et al [67], 2022	Medical imaging		Algorithm and alert linked to EHR^a^		Nontranslational effectiveness study		PDSA^b^ IHI^c^ model	
	Ali et al [68], 2020	Anesthetic		Prefilled electronic form and alerts		Nontranslational effectiveness study		PDSA IHI model	
	Bernonille et al [69], 2011	Prescription		Web app and EHR CPOE^d^		Installation		Gaston framework by de Clercq et al [105]	
	Bertoni et al [70], 2019	Prescription		Electronic order set in EHR		Full implementation		PDSA IHI model	
	Cho et al [71], 2023	Falls screening tool		Algorithm linked to EHR		Nontranslational effectiveness study		PDSA	
	Ellouzi et al [64], 2017	Hospital-acquired infection pilot		Clinical dashboard		Installation		Theoretical framework for multiagent-based decision support	
	Flaherty et al [72], 2021	Asthma prescription		Algorithm linked to EHR		Nontranslational effectiveness study		PDSA	
	Hanna et al [73], 2020	Cancer clinical screening		Electronic form in EHR		Initial implementation		PDSA	
	Jemal et al [63], 2019	Clinical deterioration		Prediction modeling		Installation		Multiagent system and intuitionistic fuzzy logic	
	Khazaei et al [74], 2015	Hospital-acquired infection		Prediction modeling linked to EHR		Exploration and installation		Research and clinical framework	
	Klunk et al [75], 2021	Prescription		Electronic order set in the EHR		Nontranslational effectiveness study		PDSA IHI model	
	Leibowitz et al [76], 2018	Blood infections		Electronic order set in EHR		Nontranslational effectiveness study		PDSA	
	Maia and Lapão [77], 2017	Hospital-acquired infection		HAI-TooL^e^ and alert and surveillance		Exploration, installation, initial implementation, and full implementation		Design science research methodology	
	Simões et al [78], 2018	Prescription		HAI-Tool alerts and patient timeline		Exploration, installation, initial implementation, and full implementation		Design science research methodology	
	Sinvani et al [79], 2020	Prescription		Electronic order set in EHR		Exploration, installation, initial implementation, and full implementation		Four phases	
	Stanger et al [80], 2018	Prescription		Electronic prescription and automated form		Installation, initial implementation, and full implementation		PDSA	
	Streiff et al [81], 2012	Blood infections		CPOE		Exploration, installation, and initial implementation		TRIP^f^ model	
	Tan et al [82], 2021	Vaccination		Electronic nursing checklist		Nontranslational effectiveness study		PDSA	
**Determinant framework**									
	Bomba and Land [83], 2006	Pharmacy		Electronic prescribing		Exploration		Triangle of Dependencies Model by Sauer	
	Brunner et al [84], 2020	Medical imaging		Clinical alert		Exploration, installation, and initial implementation		Sociotechnical framework	
	Chinman et al [85], 2019	Opioid screening		Electronic dashboard		Initial implementation and full implementation		Consolidated Framework for Implementation Research	
	Förberg et al [86], 2016	Blood infections		Electronic reminder in EHR		Installation and initial implementation		Alberta context tool	
	Geva et al [87], 2021	Blood infections		Web-based, static checklist and EHR		Installation		Five Rights of Clinical Decision Support	
	Kuo and Chang [88], 2014	Prescription		CPOE and mobile app		Installation		TAM^g^ for mobile service	
	Kawamoto et al [89], 2021	Neonatal bilirubin management app (hospital-acquired infection), atrial fibrillation stroke calculator, diabetes pharmacotherapy outcome prediction app, lung cancer screening shared decision-making app		Application within EHR-clinical guidelines, chart, and recommendations; clinical calculators embedded in EHR; application within EHR with predictive modeling; and an EHR-integrated shared decision-making screening tool		Exploration, installation, and initial implementation		Exploration, Preparation, Implementation, and Sustainment Framework	
	Mosch et al [90], 2022	Intensive care monitoring and prediction modeling		Tablet computer–based remote patient monitoring and electronic chart		Installation		Consolidated Framework for Implementation Research	
	Patapovas et al [91], 2013	Prescription		Electronic template and alerts in EHR		Initial implementation		TAM 2^h^	
	Sheehan et al [92], 2013	Medical imaging		Clinical prediction rules and guidelines		Exploration		Sociotechnical framework	
	Shelley et al [93], 2018	Dental		Electronic chart system and workﬂow		Initial implementation		Adaptation and modiﬁcation framework by Stirman	
	Stablein et al [94], 2003	Prescription		CPOE		Exploration		CPOE readiness assessment tool	
	van Engen-Verheul et al [95], 2016	Cardiology		EHR and clinical decision support guidelines		Installation		Heuristic principles by Zhang et al [106]	
	Wilk et al [96], 2013	Asthma		Prediction modeling		Installation		Task-based support architecture and organization-based multiagent system engineering method	
**Evaluation framework**									
	Fuller et al [97], 2020	Discharge		Dashboard		Installation and initial implementation		RE-AIM^i^ framework	
	Melnick et al [98], 2022	Prescription		Electronic order set in EHR		Installation and initial implementation		RE-AIM framework	
**Theory**									
	Blandford et al [99], 2022	Ophthalmology screening and referral		Telehealth and artificial intelligence		Nontranslational effectiveness study		Normalization process theory	
**Model and determinant framework**									
	Afshar et al [100], 2023	Prescription		Natural language processing, artificial intelligence, and alerts		Initial implementation		PDSA and Consolidated Framework for Implementation Research	
**Model and evaluation framework**									
	Jacobsohn et al [101], 2022	Falls screening tool		Prediction modeling and alert in EHR		Exploration and installation		Process model by Jacobsohn et al [101] and RE-AIM framework	
	Mehanni et al [65], 2019	Prescription		Electronic order set in the EHR		Exploration, installation, and initial implementation		PDSA cycles and World Health Organization’s Systems Thinking Framework	
**Evaluation framework and determinant framework**									
	Paulsen et al [102], 2021	Nutrition		Electronic report generated on a website portal		Nontranslational effectiveness study		RE-AIM framework with Consolidated Framework for Implementation Research	
	Vasudevan et al [66], 2020	Cancer digital case management system		Clinical prediction rules and guidelines		Full implementation		Consolidated Framework for Implementation Research and RE-AIM framework	
**Theory and determinant framework**									
	Fujimori et al [103], 2022	Cardiology emergency department prediction modeling		Natural language processing algorithm in EHR		Exploration and installation		Unified Theory of Acceptance and Use of Technology and Consolidated Framework for Implementation Research	
	Hsiao and Chen [104], 2016	Not stated		Clinical decision support guidelines		Installation		Active theory with extended TAM	

^a^EHR: electronic health record.

^b^PDSA: Plan-Do-Study-Act.

^c^IHI: Institute for Healthcare Improvement.

^d^CPOE: computerized provider order entry.

^e^HAI-Tool: Healthcare-associated Infections Tool.

^f^TRIP: translating research into practice.

^g^TAM: technology acceptance model.

^h^TAM 2: technology acceptance model 2.

^i^RE-AIM: Reach, Effectiveness, Adoption, Implementation, and Maintenance.

### Theories, Models, and Frameworks Used to Inform CDSS Implementation in Full-Text Published Studies

**Figure 2:**
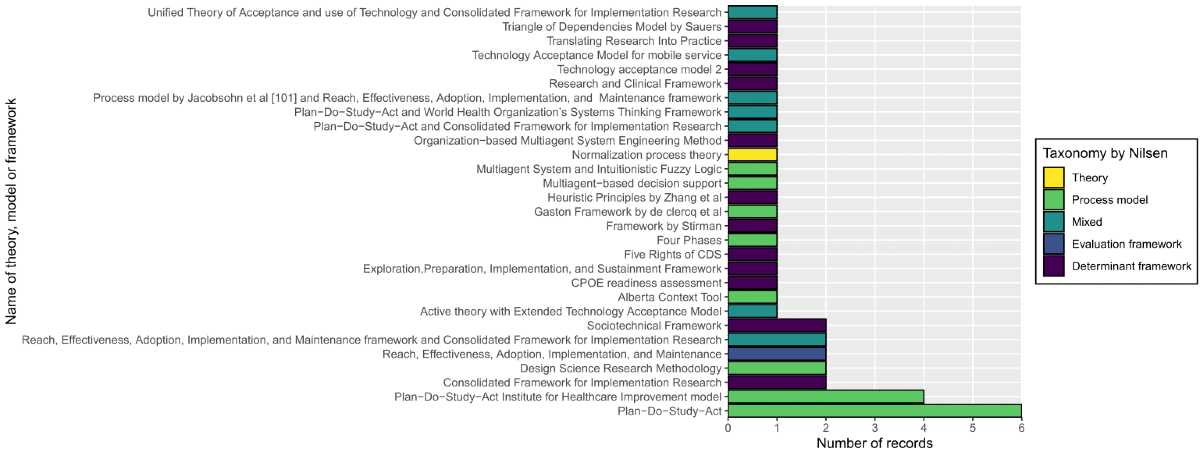
Theories, models, and frameworks used in implementing computerized clinical decision support system (CDSS) in tertiary health care settings from the included full-text records (n=42). CDS: clinical decision support; CPOE: computerized provider order entry.

The following approaches were used to inform CDSS implementation in the order of frequency: models (18/42, 43%), determinant frameworks (14/42, 33%), mixed approaches (7/42,17%), evaluation framework (2/42, 5%), and theory (1/42, 2%; Figure 2 [105,106]).

When a single approach was used, which was the case in most studies (35/42, 83%), a range of theories, models, or frameworks was used (21/35, 60% distinct theories, models, or frameworks). Of those, the Plan-Do-Study-Act (PDSA) cycle model (10/35, 29%) was the most used, followed by the CFIR (2/35, 6%) and Reach, Effectiveness, Adoption, Implementation, and Maintenance evaluation framework (2/35, 6%), with the remaining approaches very varied, as shown in Figure 2.

Most studies (24/42, 57%) reported the rationale for using a theory, model, or framework. The rationales reported included the formulation of knowledge or research questions [69,97]; addressing implementation factors leading to the use of CDSSs; and developing an implementation plan, including addressing modifications to an intervention [92,93,100]. Others cited the existing use or development of a theory, model, or framework [85,86,104] and the use of theory in aiding the scalability of intervention in different settings [74,77]. Some studies reported using a theory, model, or framework to generate possible solutions to a clinical need or to explore a complex system [84,96,102], whereas others cited synergy of the proposed framework with other implementation tools such as the Expert Recommendations for Implementing Change (ERIC) list of strategies [90].

The strengths and limitations of theories, models, and frameworks used for implementation were not reported in most studies. Most studies (28/42, 67%) did not report the strengths of a specific theory, model, or framework used, and 86% (36/42) of the studies did not report the limitations of applying a specific theory, model, or framework.

Studies that reported on the strengths of applying a specific theory, model, or framework referred to the lessons learned [80,90,97], implementation plans developed [102], ability to address barriers and facilitators for implementation within the intervention [92,94], adaptability of an intervention or increased scale-up [72,74,93], buy-in from stakeholders [65,76], use of interdisciplinary knowledge [64], and synergy with other implementation tools such as the ERIC [100].

Studies that reported the limitations of applying a specific theory, model, or framework referred to the need for more “finely tuned models” [83], including iterative validation of tools such as the CFIR-ERIC implementation tool [90] or iterating the scoring of items on a CPOE readiness assessment tool [94]; difficulty characterizing determinants that were too interdependent using a determinant framework (eg, the sociotechnical model) [92]; and unfamiliarity with theory, model, or framework processes (such as the use of quality improvement processes, eg, PDSA cycles by clinicians) [65].

### Findings From Study Abstracts

The use of 19 different theories, models, and frameworks was reported in 39 included abstracts (Multimedia Appendix 5 [107-145]). Of these 39 abstracts, 4 (10%) used a theory, model, or framework but lacked sufficient detail to classify this using the taxonomy by Nilsen [30]. Of the abstracts that could be classified, the majority used models (27/33, 81%), with the PDSA cycle model being the most used (19/33, 58%).

## Discussion

### Principal Findings

This scoping review identified that the distinct theories, models, and frameworks (26/42, 62%) used in the implementation of CDSS in tertiary health care settings in included full-text studies were informed by implementation science and also by quality improvement and information technology fields. Although this highlighted the use of varying perspectives to inform CDSS implementation activities across the included full-text studies, this review indicated that process models were used most frequently.

Models assist with the practical application of an evidence-based intervention, within a system, by describing implementation-specific activities in practice [30,146]. In total, 56% (45/81) of the theories, models, and frameworks used in both full text and abstracts included in our review were categorized as models based on the taxonomy by Nilsen [30]. The frequent use of models is not unexpected, given that the use of such models is common in quality improvement, which is familiar to many working in hospital settings [147]. Process models, in particular, break implementation activities into smaller steps, making them useful in complex hospital settings with diverse stakeholders, psychosocial and behavioral influences, and resource constraints.

Despite advantages, solely using models to inform implementation approaches may hinder the long-term sustainment, monitoring, and iteration of CDSS innovations within hospital systems. Models alone, particularly simplified process models (ie, PDSA), do not typically examine the causal mechanisms for CDSS implementation or systematically investigate implementation factors, including barriers or facilitators [30,148]. A failure to account for implementation factors and mechanisms may misinform the planning and resource allocations required for subsequent implementation strategies and evaluations. In addition, without appropriate consideration of the barriers and facilitators to implementation (by conducting a context assessment using a determinants framework), the likelihood of implementation success may be adversely impacted.

Appropriate consideration of barriers and facilitators is particularly important for intersectional and multifaceted interventions comprising CDSSs and related digital innovations in health care settings. This includes integrated electronic medical record systems, which often incorporate a range of modules to inform prescribing practices, categorize patient risks, and chart vital signs. The application and use of these innovations typically require a range of system-level modifications. This could require engagement with various stakeholders within the hospital and the broader health care delivery system. These stakeholders may include informatics and health systems professionals, a range of clinical end users (such as nursing, allied health, and medical staff), and the provision of ongoing technical support [16]. Consequently, the sustainable ongoing adoption and appropriate use of CDSS requires targeted ongoing investment in upskilling staff, added labor, and the management of data outputs and related assets [24,25]. Theories and frameworks applicable to translational medicine, human factors, and implementation science were borne from the need to address this complexity, and their use in facilitating the uptake of digital innovations within health care remains relatively untapped [149].

This review highlighted that over half (29/44, 65%) of the included papers (full texts and abstracts) that only used a model reported using a quality improvement PDSA cycle to inform their implementation of a CDSS.

Although PDSA cycles may be beneficial for tailoring implementation activities within local health system settings, relying solely on quality improvement–informed processes may limit the effective implementation of novel or multisystem interventions (a frequent characteristic of CDSS tools) within hospital settings. Quality improvement initiatives are often small scale and context specific, which may undermine scalability to other settings [150,151]. Of note was the wide application of PDSA cycles within hybrid effectiveness-implementation studies. These experiments adopted PDSA cycles to inform implementation strategies within a protocolized methodology as part of an effectiveness trial. Given that this accounted for 21% (9/42) of the included studies, it is notable that various CDSS innovations are still undergoing effectiveness testing and may not yet be ready for systematic translation. Furthermore, the frequent use of PDSA cycles in both pretranslational and translational studies suggests the need to move beyond quality improvement models to capture implementation outcomes in translational implementation studies.

This review found that over half of the authors reported the rationale for using a theory, model, or framework (24/42, 57%), suggesting contextual knowledge in their approach. Conversely, less than half (18/42, 43%) did not do this, which may be owing to the potential time, expertise, and training required to apply the suite of these approaches. This cognitive burden may be considered impractical by those on the front lines of health care who must respond rapidly to circumstances that could endanger lives [38]. However, knowing the strengths of a particular theory, model, or framework can assist in building a case for use. It may help develop knowledge of practically applying theory to enhance practice [38]. Our findings corroborated previous research that using theories, models, and frameworks assists in facilitating implementation strategies, fostering engagement with stakeholders, and creating generalizable knowledge [46,152].

The limitations of applying theories, models, and frameworks in practice should also be investigated. Even specified preimplementation deterministic frameworks may not be applied with fidelity, making it difficult to establish the effect of a theoretical viewpoint and related strategies on implementation efforts. Interventions are also influenced by the contextual settings, with process evaluations demonstrating how theory-informed implementation may stray from the devised plan and be affected by contextual factors or determinants [153,154]. Failure to record this information may lead to duplication of implementation efforts in different hospital settings, which could otherwise be prevented [151,154]. Reporting the practical constraints of applying theories, models, or frameworks to inform CDSS implementation activities may benefit future implementers whether they are clinicians or researchers.

### Implications for Practice and Research

This review found that only 7 (17%) of the 42 included full-text studies attempted to use a mix of theories, models, or frameworks. This is despite recommendations that a combination of theory, model, and framework-informed approaches may be needed to target a range of contextual domains throughout the implementation process for complex interventions such as CDSS adoption [40,149]. Using theoretically informed methodologies may also assist in reducing the duplication of research and implementation efforts, which may improve the current lag of CDSS implementation and uptake in hospital settings [151]. Theories and frameworks, in addition to models, allow the systematic evaluation of discrete implementation science principles across a range of contextually specific health care settings. This allows for generalizable recommendations for the uptake of evidence-based interventions across such settings [30,39,146]. Specifically, it has been suggested that a theory or a deterministic framework should be used in conjunction with familiar process models and quality improvement–informed strategies such as PDSA cycles [115,116,155]. Thus, using a quality improvement–informed process model will likely provide recognizable guidance for health care professionals in implementing CDSSs in practice, but the addition of a deterministic framework may assist in informing generalizable implementation across settings. The use of both a quality improvement process model and a deterministic framework may assist in bridging the often-siloed fields of implementation science and improvement practice to create engaging implementation plans for decision makers and policy makers [156].

### Methodological Considerations

This scoping review had several methodological limitations. Although peer-reviewed abstracts were included in this review, little information apart from the type of theory, model, or framework could be identified from the included abstracts. The study settings were also limited to tertiary health care contexts to address the study aim; therefore, the findings of this review are not likely to be generalizable to other settings.

Studies that described the development of CDSS were also excluded from this review. However, given the increasing use of co-design to develop and implement stakeholder-approved CDSS tools, future reviews examining intervention development studies informed by implementation science theories, models, or frameworks may offer additional insights into the systematic application of implementation principles in health care settings.

### Conclusions

This review of theories, models, and frameworks to inform implementation planning and adoption of CDSS interventions in tertiary health care settings identified the frequent use of quality improvement methods, classified as models, to inform implementation activities, evaluation, and experimental studies. Although pragmatically beneficial in sustaining improvements over certain periods within specific clinical or service delivery contexts, non–theory-informed and nondeterministic frameworks typically lack a multifactorial system-wide approach. Non–theory-informed and nondeterministic frameworks may also not be well suited to multifaceted, intersectional, and complex interventions such as CDSSs and related digital innovations, particularly within an acute health care context. Theories, models, and frameworks were borne from the need to address this complexity, but their potential to accelerate the adoption of digital breakthroughs in health care remains relatively untapped. Furthermore, this review highlighted the lack of consolidated or standardized reporting to describe the application of implementation approaches for CDSS interventions. Reporting implementation activities may benefit from the wider use of consolidated guidelines such as the Standards for Reporting Implementation Studies [157]. However, further work may be required to evaluate why guidelines such as Standards for Reporting Implementation Studies are not commonly used, particularly within the digital health context. Consequently, there is a need for ongoing work to facilitate a more systematic and transparent reporting of implementation strategies to diffuse what works versus what does not work in the digital health field.

## Appendix

Multimedia Appendix 1 PRISMA-ScR (Preferred Reporting Items for Systematic Reviews and Meta-Analyses extension for Scoping Reviews) checklist.

Multimedia Appendix 2 Search strategy.

Multimedia Appendix 3 Definitions and elaborations document.

Multimedia Appendix 4 Primary search PRISMA (Preferred Reporting Items for Systematic Reviews and Meta-Analyses) flow diagram.

Multimedia Appendix 5 Abstracts included.

## Abbreviations

CDSS: computerized clinical decision support system
CFIR: Consolidated Framework for Implementation Research
CPOE: computerized provider order entry
ERIC: Expert Recommendations for Implementing Change
PDSA: Plan-Do-Study-Act
PRISMA-ScR: Preferred Reporting Items for Systematic Reviews and Meta-Analyses extension for Scoping Reviews
