# Supplementary material for: Using Theories, Models, and Frameworks to Inform Implementation Cycles of Computerized Clinical Decision Support Systems in Tertiary Health Care Settings: Scoping Review
Source: J Med Internet Res. 2023 Oct 18;25:e45163. doi: 10.2196/45163 (PMC10620641; doi:10.2196/45163)
Supplement: Multimedia Appendix 2 [file jmir_v25i1e45163_app2.docx]

Multimedia Appendix 2

SEARCH STRATEGY PRIMARY

What are the implementation theories, models and frameworks that have been utilized in implementing clinical decision support systems in tertiary healthcare settings including hospitals?

**Platform** PubMed

**Databases** Embase via Elsevier, Cumulative Index to Nursing and Allied Health Literature (CINAHL) via EBSCO, Scopus, Web of science

**Search date coverage** No date limit

**Search strategy**

1. "Health Plan Implementation"[MeSH Terms]
2. "Implementation Science"[MeSH Terms]
3. "Health Plan Implementation"[Title/Abstract]
4. "Implementation Science"[Title/Abstract]
5. "implement*"[Title/Abstract])
6. OR/1-5
7. "framework*"[Title/Abstract]
8. "model*"[Title/Abstract]
9. "theor*"[Title/Abstract]
10. "Mapping"[Title/Abstract]
11. "program*"[Title/Abstract]
12. "tool*"[Title/Abstract]
13. "assessment*"[Title/Abstract]
14. OR/7-13
15. "decision support systems, clinical"[MeSH Terms]
16. "Artificial Intelligence"[MeSH Terms]
17. "clinical decision support system*"[Title/Abstract]
18. "computerised decision support system*"[Title/Abstract]
19. "computerized decision support system*"[Title/Abstract]
20. "decision support*"[Title/Abstract]
21. "cdss"[Title/Abstract]
22. "Artificial Intelligence"[Title/Abstract]
23. "AI"[Title/Abstract]
24. "reminder system*"[Title/Abstract]
25. "electronic feedback"[Title/Abstract]
26. "dashboard*"[Title/Abstract])
27. OR/ 15-26
28. "Hospitals"[MeSH Terms]
29. "hospital*"[Title/Abstract]
30. "ward*"[Title/Abstract]
31. "medical centre*"[Title/Abstract]
32. "medical center*"[Title/Abstract]
33. OR/28-32
34. 6 AND 14 AND 27 AND 33

**Number of citations** 1369

Search conducted 17/05/2021

**­Notes**

1. PubMed used rather than MEDLINE based on advice from a medical librarian with scoping review experience. Furthermore, this was the database used to build the initial search strategy.
2. Although, most other searches within this field included PsychInfo as a database this was excluded from this search strategy as initial searches only revealed two results which were included from other database searches in Scopus and Embase.
3. No date limit was set as searches showed a natural timeline of relevant articles with the introduction of terms such as ‘artificial intelligence’ around 1980’s to ‘implementation science’ within the past five years. Indeed, the earliest articles were from the 1980’s to the present.
4. Line 1 "Health Plan Implementation"[MeSH Terms] was added as PubMed included a MESH term that aligned with the key word of implementation. It was introduced in 1981 and was described as ‘those actions designed to carry out recommendations pertaining to health plans or programs’ which could include implementation strategies related to a theory, model or framework
5. Line 2 "Implementation Science"[MeSH Terms] was added to capture implementation science articles. The MeSH term was only included in 2019 and is described as the study of methods to promote the adoption and integration of evidence-based practices, interventions and policies into routine health care and public health settings.
6. Lines 3-5 were included to capture all the data as MESH terms such as ‘implementation science’ only started being utilised in 2019 in the PubMed database and potential articles could be missed.
7. Line 5 "implement*"[Title/Abstract]) was added to capture “implementation”, “implemented”, “implements” and other similar ending terms.
8. Lines 7-9 were based on Nilsen (2015) search strategy examining implementation theories, models and frameworks within the literature.
9. Line 7 ("framework*"[Title/Abstract] was added to capture “frameworks” and other ending similar terms.
10. Line 8 "model*"[Title/Abstract] was added to capture “models” and other similar ending terms.
11. Line 9 "theor*"[Title/Abstract] was added to capture “theories”, “theoretical”, “theorised” and other similar ending terms.
12. Lines 10-13 were based on a combination of search strategies from Betty et al. (2015) and Powell et al. (2012) examining implementation science. Furthermore, table 6 from Birken et al. (2017) was consulted and key terms from the names of utilised theories, models and frameworks was added to the search strategy.
13. Line 10 "Mapping"[Title/Abstract] was specifically added as PubMed included many irrelevant results and irrelevant endings with “Map*”.
14. Line 11 “program*"[Title/Abstract] was added to capture “programs”, “programmed” or “programming” and other similar ending terms.
15. Line 12 "tool*" was added to capture “tools”, “toolkit’ and other similar ending terms. This word was added as some existing theories, models or frameworks have tools to be used practically in interventions. For example, the CFIR (Consolidated Framework for Implementation Research) have developed interview guide tools.
16. Line 13 "assessment*"[Title/Abstract]) was added to included “assessments” and other similar ending terms. This term was added as it could capture assessments conducted as a part of a theory, model or framework.
17. Lines 15-26 were based on a combination of search strategies from Betty et al. (2015), Sutton et al. (2020) and Kilsdonk et al. (2011) examining the literature on clinical decision support systems.
18. Line 15 "decision support systems, clinical"[MeSH Terms] was added as a MeSH term. It was introduced as a MeSH term in 1998 and described as computer-based information systems used to integrate clinical and patient information and provide support for decision-making in patient care.
19. Line 16 "Artificial Intelligence"[MeSH Terms] was added as a MeSH term. It was introduced as a MeSH term in 1986 and described as the theory and development computer systems which perform tasks that normally require human intelligence.
20. Line 17 "clinical decision support system*"[Title/Abstract] was added to capture anything missed by the MeSH headings and to capture “clinical decision support systems” or other similar ending terms
21. Line 18 and 19 "computerised decision support system*"[Title/Abstract] and "computerized decision support system*"[Title/Abstract] were used specifically to capture language variation. A wildcard wasn’t used on advice from medical librarian and for ease of use when transferring the search to other databases.
22. Line 18"computerised decision support system*"[Title/Abstract] was added to capture “computerised decision support systems” and other similar ending terms.
23. Line 19 "computerized decision support system*"[Title/Abstract] was added to capture “computerized decision support systems” and other similar ending terms.
24. Line 20 "decision support*"[Title/Abstract] was used in Betty et al. (2015) search strategy and it was added to capture “decision supports” and other similar ending terms.
25. Line 21"cdss"[Title/Abstract] was added to capture any articles using abbreviations of “clinical decision support system” or “computerised decision support system”. Phrase and title abstract searching were used to keep the search relevant.
26. Line 22 "Artificial Intelligence"[Title/Abstract] was added to capture any articles missed by the MeSH headings.
27. Line 23 “"AI"[Title/Abstract] was added to capture any articles using the abbreviation of “artificial intelligence. Phrase and title abstract searching were used to keep the search relevant.
28. Line 24 "reminder system*"[Title/Abstract] was added to capture examples of common CDSS and to capture “reminder systems” or other similar ending terms.
29. Line 25 "electronic feedback"[Title/Abstract] was specifically added to capture examples of common CDSS. PubMed included many irrelevant results and irrelevant endings with “electronic feedback *”.
30. Line 26 "dashboard*"[Title/Abstract]) was added to capture examples of common CDSS and to capture “dashboards” or other similar ending terms.
31. Line 28 ("Hospitals"[MeSH Terms] was added to capture the hospital setting. The term describes institutions with an organized medical staff which provide medical care to patients.
32. Line 29 ("hospital*"[Title/Abstract] was added to capture any terms missed from the MeSH heading and to capture “hospitals”, “hospitalisations” and other similar ending terms.
33. Line 30 and Line 31 "medical centre*"[Title/Abstract] and "medical center*"[Title/Abstract])) were used specifically to capture language variation. A wildcard wasn’t used on advice from medical librarian and for ease of use when transferring the search to other databases. A MeSH term for medical centre could not be found with the closest MeSH term being “academic medical centre” which may bias the results.
34. No search restrictions (i.e. English only, human not animal) applied.

**Platform** Embase®

**Databases** EMBASE via Elsevier

**Search date coverage** No date limit

**Search strategy**

1. **'health care planning'**/exp
2. 'health care planning'
3. **'implementation science'**/exp
4. 'implementation science'
5. 'health care planning’: ab,ti
6. 'implementation science’: ab,ti
7. 'implement*’: ab,ti
8. OR/1-7
9. 'framework*’: ti,ab
10. 'model*’: ti,ab
11. 'theor*’: ti,ab
12. 'mapping’: ti,ab
13. 'program*’: ti,ab
14. 'tool*’: ti,ab
15. 'assessment*’: ti,ab
16. OR/9-15
17. **'clinical decision support system'**/exp
18. **'artificial intelligence'**/exp
19. 'clinical decision support system*’: ti,ab
20. 'computerised decision support system*’: ti,ab
21. 'computerized decision support system*’: ti,ab
22. 'decision support*’: ti,ab
23. 'cdss’: ti,ab
24. 'artificial intelligence’: ti,ab
25. 'ai’: ti,ab
26. 'reminder system*’: ti,ab
27. 'electronic feedback’: ti,ab
28. 'dashboard*’: ti,ab
29. OR/17-28
30. **'hospital'/exp**
31. **'medical centers'/exp**
32. **'medical center'/exp**
33. 'hospital*’: ti,ab
34. 'ward*’: ti,ab
35. 'medical centre*’: ti,ab
36. 'medical center*’: ti,ab
37. OR/30-36
38. 8 AND 16 AND 29 AND 37
39. medline]/lim
40. NOT/39

**Number of citations** 1145

Search conducted 17/05/2021

**­Notes**

1. Line 1 **'health care planning'**/exp was included as an emtree term to be consistent with Pubmed search. The term was added in 1987 and defined along the lines of community health planning; health and welfare planning; health plan implementation; health planning; health planning guidelines; health planning organisations, health planning support etc.
2. Line 2 'health care planning' was added to capture any terms missed by the emtree heading and to be consistent with the Pubmed search.
3. Line 3 **'implementation science'**/exp was added to an emtree term. The term was added in 2019 and describes implementation research and implementation scientific research.
4. Line 4 'implementation science' was added as a phrase search to capture any missing terms from emtree headings and title/abstract search. Around 7184 results were found compared to around 4093 results from emtree headings and title/abstract search of line 3 and 5.
5. Line 17 **'clinical decision support system'**/exp was added as an exploded emtree heading. The term was added in 2016 and described CDS system (clinical decision support system); clinical decision support (CDS) system; clinical decision support systems; clinician decision support system and decision support systems, clinical.
6. Line 18 **'artificial intelligence'**/exp was added as an exploded emtree term. The term was added in 1974 and describes a branch of computer science in which machines are made to perform functions similar to the intellectual functions of the human mind, such as learning and reasoning.
7. Lines 19-22 were included to be consistent to PubMed search and to capture articles missed by the emtree heading.
8. Lines 20 and 21 'computerised decision support system*’: ti,ab and 'computerized decision support system*’: ti,ab were added to be consistent to PubMed search and to account for language variation.
9. Line 22 'decision support*’: ti,ab although there is an emtree term for ‘decision support’ this was not added to the search as **'clinical decision support system'** fell under this emtree term and was more specific to the search being conducted.
10. Line 23-line 28 was included to be consistent to pubmed search and to capture articles missed by the emtree heading.
11. Line 30 **'hospital'/exp** was added as an exploded emtree heading. The term was added in 1974 and describes many different hospitals settings such as emergency hospital; environment, hospital; hospital data; hospital environment; hospital establishment; regional hospital; state hospital; voluntary hospital etc.
12. Line 31 **'medical centers'/exp** was a candidate emtree term that was included to capture language variation and any missing/specific articles.
13. Line 32 **'medical center'/exp** was a candidate emtree term that was included to capture language variation and any missing/specific articles.
14. Line 33-36 included to stay consistent with Pubmed searches and in particular lines 35 and 36'medical centre*’: ti,ab and 'medical center*’: ti,ab were added to account for language variation.
15. Line 38 medline]/lim was added on recommendation from medical librarian with scoping review experience to exclude Medline results. This removes potential duplicates from the PubMed search and allows for potential new articles to be captured.
16. No other search restrictions (i.e. English only, human not animal) applied.

**Platform** EBSCO

**Databases** Cumulative Index to Nursing and Allied Health Literature (CINAHL)

**Search date coverage** No date limit

**Search strategy**

1. MH "Health Facility Planning+"
2. MH "Implementation Science"
3. TI “implement*”
4. AB “implement*”
5. TI “Health Facility planning”
6. AB “Health Facility planning”
7. TI “Implementation Science”
8. AB “Implementation Science”
9. OR/1-8
10. TI framework*
11. AB framework*
12. TI model*
13. AB model*
14. TI theor*
15. AB theor*
16. TI Mapping
17. AB Mapping
18. TI program*
19. AB program*
20. TI tool*
21. AB tool*
22. TI assess*
23. AB assess*
24. OR/10-23
25. MH "Decision Support Systems, Clinical"
26. MH "Artificial Intelligence+"
27. TI "clinical decision support system*”
28. AB "clinical decision support system*"
29. TI "computerised decision support system*"
30. AB "computerised decision support system*"
31. TI "computerized decision support system*"
32. AB "computerized decision support system*"
33. TI "decision support*"
34. AB "decision support*"
35. TI “cdss”
36. AB “cdss”
37. TI "Artificial Intelligence"
38. AB "Artificial Intelligence"
39. TI “AI”
40. AB “AI”
41. TI "reminder system*"
42. AB "reminder system*"
43. TI "electronic feedback"
44. AB "electronic feedback"
45. TI “dashboard*”
46. AB “dashboard*”
47. OR/ 25-26
48. MH "Hospitals+"
49. TI “hospital*”
50. AB “hospital*”
51. TI “ward*”
52. AB “ward*”
53. TI "medical centre*"
54. AB "medical centre*"
55. TI "medical center*"
56. AB "medical center*"
57. OR/48-56
58. 9 AND 24 AND 47 AND 57

**Limiters** - Full Text

**Expanders** - Apply equivalent subjects

**Search modes** - Boolean/Phrase

**Number of citations** 635

Search conducted 17/05/2021

**­Notes**

1. Line 1 MH "Health Facility Planning+" was added as an exploded major heading to be consistent with the terms ‘health plan implementation’ and ‘health care planning’ in the PubMed and Embase searches. It is described as area-wide planning for healthcare institutions on the basis of projected consumer needs and demand and includes setting specific hospital planning which describes strategy of building of hospitals or a specific hospital unit on the basis of projected consumer needs and demand.
2. Line 2 MH "Implementation Science" was added as a major heading to be consistent with Pubmed searches and the term describes the study of promoting evidence-based practices and policies and their adoption.
3. Line 3-4 TI “implement*” and AB “implement*” was added to capture missing terms from major heading searches and to be consistent with PubMed search strategy.
4. Lines 5-6 TI “Health Facility planning” and AB “Health Facility planning” was added to capture missing terms from the major heading search.
5. Lines 7-8 TI “Implementation Science” and AB “Implementation Science” was added to capture missing terms from major heading searches and to be consistent with PubMed search strategy.
6. Lines 10-23 was added to be consistent with the PubMed search strategies.
7. Line 25 MH "Decision Support Systems, Clinical" was added as a major heading to be consistent with PubMed and Embase terms and describes systems that integrate clinical data and patient data to support the decisions made by the clinicians. These systems contain numerous databases of information such as drug interactions and contraindications, treatment costs, clinical practice guidelines, and critical paths.
8. Line 26 MH "Artificial Intelligence+" was added as an exploded major heading to be consistent with the PubMed search. It describes the capacity of a device to perform functions normally associated with human intelligence, such as reasoning, learning and self-improvement and includes major headings for the uses of AI such as expert systems, knowbots, knowledge bases, machine learning, natural language processing, neural networks (computer), robotics and blockchain.
9. Line 37-34 was added to stay consistent to PubMed search and capture any missing terms from the major heading search for line 25 MH "Decision Support Systems, Clinical".
10. Line 33-34 TI "decision support*" and AB "decision support*" was specifically included to stay consistent to PubMed searches and as there was no major heading for “decision support” with the closest major heading being line 25 MH "Decision Support Systems, Clinical".
11. Line 37-40 was added to be consistent to the PubMed search and to capture any missing terms from the major heading search for line 26 MH "Artificial Intelligence+"
12. Line 29-32 were added to be consistent to PubMed searches and to capture any language variation.
13. Line 41-46 was added to be consistent to PubMed search and to capture articles missed by the major heading searches.
14. Line 48 MH "Hospitals+" was added as an exploded major heading. The term describes an institution that provides care and treatment for the sick and injured. The term also includes other major headings such as hospitals, community; hospitals, private; hospitals, public; hospitals, rural; hospitals, special; hospitals; urban; magnet hospitals and housing for the elderly.
15. Line 49-52 were added to be consistent to PubMed searches and to capture any missing terms or articles from line 48 major heading search MH "Hospitals+"
16. Line 53-56 were added to be consistent to PubMed searches and to account for language variation.
17. Line 53-56 was added as like PubMed the closest major heading was ‘academic medical centres’ which may have biased the results.
18. No other search restrictions (i.e. English only, human not animal) applied.

**Platform** Elsevier

**Databases** Scopus

**Search date coverage** No date limit

**Search strategy**

1. INDEXTERMS (“Health Plan Implementation”)
2. INDEXTERMS (“Implementation Science”)
3. TITLE-ABS (“implement*”)
4. TITLE-ABS (“Health Plan Implementation”)
5. TITLE-ABS (“Implementation Science “)
6. OR/1-5
7. TITLE-ABS (“framework*”)
8. TITLE-ABS (“model*”)
9. TITLE-ABS (“theor*”)
10. TITLE-ABS (“Mapping”)
11. TITLE-ABS (“program*”)
12. TITLE-ABS (“tool*”)
13. TITLE-ABS (“assess*”)
14. OR/7-13
15. INDEXTERMS (“decision support systems, clinical”)
16. INDEXTERMS (“Artificial Intelligence”)
17. TITLE-ABS (“clinical decision support system*”)
18. TITLE-ABS (“computerised decision support system*”)
19. TITLE-ABS (“computerized decision support system*”)
20. TITLE-ABS (“decision support*”)
21. TITLE-ABS (“cdss”)
22. TITLE-ABS (“Artificial Intelligence”)
23. TITLE-ABS (“AI”)
24. TITLE-ABS (“reminder system*”)
25. TITLE-ABS (“electronic feedback”)
26. TITLE-ABS (“dashboard*”)
27. OR/15-26
28. INDEXTERMS (“Hospital”)
29. TITLE-ABS (“hospital*”)
30. TITLE-ABS (“ward*”)
31. TITLE-ABS (“medical centre*”)
32. TITLE-ABS (“medical center*”)
33. OR/28-32
34. 6 AND 14 AND 27 AND 33

**Number of citations** 1892

Search conducted 17/05/2021

**­Notes**

1. Line 1 INDEXTERMS (“Health Plan Implementation”) was added to be consistent with the PubMed search
2. Line 2 INDEXTERMS (“Implementation Science”) was added to be consistent with the PubMed search
3. Lines 3-5 was added to be consistent with the PubMed search and to capture any missing data from the Index terms searches in line 1 and line 2.
4. Line 7-13 was added to be consistent with the PubMed search
5. Line 15 and line 16 INDEXTERMS (“decision support systems, clinical”) and INDEXTERMS (“Artificial Intelligence”) was added to be consistent with the PubMed search
6. Line 17-26 was added to be consistent with the PubMed search and to capture any missing data from the Index terms searches in line 15 and line 16.
7. Line 18 and 19 TITLE-ABS (“computerised decision support system*”) and TITLE-ABS (“computerized decision support system*”) was added to account for language variation
8. Line 28 INDEXTERMS (“Hospital”) was added to be consistent with the PubMed search
9. Line 30-31 was added to be consistent with the PubMed search and to capture any missing data from the Index terms searches in line 28.
10. Line 31 and 32 TITLE-ABS (“medical centre*”) and TITLE-ABS (“medical center*”) was added to account for language variation
11. No other search restrictions (i.e. English only, human not animal) applied.

**Databases** Web of Science

**Search date coverage** No date limit

**Search strategy**

**Number of citations** 954

1. TS= (“Health Plan Implementation”)
2. TS= (“Implementation Science”)
3. TI= (“Health Plan Implementation”)
4. AB= “Health Plan Implementation”
5. TI= “Implementation Science”
6. AB= “Implementation Science”
7. TI= “implement*”
8. AB= “implement*”
9. OR/1-8
10. TI= “framework*”
11. AB= “framework*”
12. TI= “model*”
13. AB= “model*”
14. TI= “theor*”
15. AB= “theor*”
16. TI= “Mapping”
17. AB= “Mapping”
18. TI= “program*”
19. AB= “program*”
20. TI= “tool*”
21. AB= “tool*”
22. TI= “assess*”
23. AB= “assess*”
24. OR/10-23
25. TS= “clinical decision support system*”
26. TS= “Artificial Intelligence”
27. TI= “clinical decision support system*”
28. AB= “clinical decision support system*”
29. TI= “computerised decision support system*”
30. AB= “computerised decision support system*”
31. TI= “computerized decision support system*”
32. AB= “computerized decision support system*”
33. TI= “decision support*”
34. AB= “decision support*”
35. TI= “cdss”
36. AB= “cdss”
37. TI= “Artificial Intelligence”
38. AB= “Artificial Intelligence”
39. TI= “AI”
40. AB= “AI”
41. TI= “reminder system*”
42. AB= “reminder system*”
43. TI= “electronic feedback”
44. AB= “electronic feedback”
45. TI= “dashboard*”
46. AB= “dashboard*”
47. OR/25-46
48. TS= “Hospital”
49. TI= “hospital*”
50. AB= “hospital*”
51. TI= “ward*”
52. AB= “ward*”
53. TI= “medical centre*”
54. AB= “medical centre*”
55. TI= “medical center*”
56. AB= “medical center*”
57. OR/48-56
58. 9 AND 14 AND 27 AND 33

Search conducted 29/04/2021

**Notes**

1. Although, initial searches were conducted in IEEE-explore and ACM two computer science and technology databases the searches were difficult to conduct or navigate with wild card restrictions/key word restrictions and too many results. Thus, Web of science was to capture technology and computer science-based articles on recommendation from a medical librarian with experience in conducting scoping reviews.
2. Line 1 and line 2 TS= ("Health Plan Implementation") and TS= ("Implementation Science") were topic sentences added to be consistent with PubMed and other searches.
3. Line 4-8 was added to be consistent with the PubMed search and to capture any missing data from the topic searches in line 1 and line 2.
4. Line 10-23 added to be consistent with the PubMed search
5. Line 25 and 26 TS=” clinical decision support system*” and TS=” Artificial Intelligence” topic searches was added to be consistent with PubMed and other searches examining CDSS and artificial intelligence
6. Line 27-46 was added to be consistent with the PubMed search and to capture any missing data from the topic searches in line 25 and line 26.
7. Lines 29-32 was added to capture language variation
8. Line 48 TS=” Hospital” topic search was added to be consistent with the PubMed and other search strategies
9. Line 49-56 was added to be consistent with the PubMed search and to capture any missing data from the topic searches in line 48
10. Line 53-56 was added to capture language variation
11. No other search restrictions (i.e. English only, human not animal) applied.

# SEARCH STRATEGY Updated

Search conducted 22 May 2023

**Platform** PubMed

**Databases** Embase via Elsevier, Cumulative Index to Nursing and Allied Health Literature (CINAHL) via EBSCO, Scopus, Web of science

**Search date coverage** No date limit

**Search strategy**

1. "Health Plan Implementation"[MeSH Terms]
2. "Implementation Science"[MeSH Terms]
3. "Health Plan Implementation"[Title/Abstract]
4. "Implementation Science"[Title/Abstract]
5. "implement*"[Title/Abstract])
6. “Adopt*” [Title/Abstract]
7. OR/1-6
8. "framework*"[Title/Abstract]
9. "model*"[Title/Abstract]
10. "theor*"[Title/Abstract]
11. "Mapping"[Title/Abstract]
12. "program*"[Title/Abstract]
13. "tool*"[Title/Abstract]
14. "assessment*"[Title/Abstract]
15. OR/7-14
16. "decision support systems, clinical"[MeSH Terms]
17. "Artificial Intelligence"[MeSH Terms]
18. "clinical decision support system*"[Title/Abstract]
19. "computerised decision support system*"[Title/Abstract]
20. "computerized decision support system*"[Title/Abstract]
21. "decision support*"[Title/Abstract]
22. "cdss"[Title/Abstract]
23. "Artificial Intelligence"[Title/Abstract]
24. "AI"[Title/Abstract]
25. "reminder system*"[Title/Abstract]
26. "electronic feedback"[Title/Abstract]
27. "dashboard*"[Title/Abstract])
28. OR/ 16-27
29. "Hospitals"[MeSH Terms]
30. "hospital*"[Title/Abstract]
31. "ward*"[Title/Abstract]
32. "medical centre*"[Title/Abstract]
33. "medical center*"[Title/Abstract]
34. OR/29-33
35. 7 AND 15 AND 28 AND 34

**­Notes**

1. PubMed used rather than MEDLINE based on advice from a medical librarian with scoping review experience. Furthermore, this was the database used to build the initial search strategy.
2. Although, most other searches within this field included PsychInfo as a database this was excluded from this search strategy as initial searches only revealed two results which were included from other database searches in Scopus and Embase.
3. No date limit was set as searches showed a natural timeline of relevant articles with the introduction of terms such as ‘artificial intelligence’ around 1980’s to ‘implementation science’ within the past five years. Indeed, the earliest articles were from the 1980’s to the present.
4. Line 1 "Health Plan Implementation"[MeSH Terms] was added as PubMed included a MESH term that aligned with the key word of implementation. It was introduced in 1981 and was described as ‘those actions designed to carry out recommendations pertaining to health plans or programs’ which could include implementation strategies related to a theory, model or framework
5. Line 2 "Implementation Science"[MeSH Terms] was added to capture implementation science articles. The MeSH term was only included in 2019 and is described as the study of methods to promote the adoption and integration of evidence-based practices, interventions and policies into routine health care and public health settings.
6. Lines 3-5 were included to capture all the data as MESH terms such as ‘implementation science’ only started being utilised in 2019 in the PubMed database and potential articles could be missed.
7. Line 5 "implement*"[Title/Abstract]) was added to capture “implementation”, “implemented”, “implements” and other similar ending terms.
8. Line 6 “adopt*” [Title/Abstract] was added to capture terms such as “adopt” “adoption” or “adopts” and others similar endings as based on expert peer reviewer comments that the field of HIT evaluation / medical informatics use terms other than implementation to describe the implementation process
9. Line 6 The MeSH term from adoption was not included as it referred to the voluntary acceptance of a child of other parents to be as one's own child, usually with legal confirmation.
10. Lines 8-10 were based on Nilsen (2015) search strategy examining implementation theories, models and frameworks within the literature.
11. Line 8 ("framework*"[Title/Abstract] was added to capture “frameworks” and other ending similar terms.
12. Line 9 "model*"[Title/Abstract] was added to capture “models” and other similar ending terms.
13. Line 10"theor*"[Title/Abstract] was added to capture “theories”, “theoretical”, “theorised” and other similar ending terms.
14. Lines 11-14 were based on a combination of search strategies from Betty et al. (2015) and Powell et al. (2012) examining implementation science. Furthermore, table 6 from Birken et al. (2017) was consulted and key terms from the names of utilised theories, models and frameworks was added to the search strategy.
15. Line 11 "Mapping"[Title/Abstract] was specifically added as PubMed included many irrelevant results and irrelevant endings with “Map*”.
16. Line 12 “program*"[Title/Abstract] was added to capture “programs”, “programmed” or “programming” and other similar ending terms.
17. Line 13"tool*" was added to capture “tools”, “toolkit’ and other similar ending terms. This word was added as some existing theories, models or frameworks have tools to be used practically in interventions. For example, the CFIR (Consolidated Framework for Implementation Research) have developed interview guide tools.
18. Line 43 "assessment*"[Title/Abstract]) was added to included “assessments” and other similar ending terms. This term was added as it could capture assessments conducted as a part of a theory, model or framework.
19. Lines 16-76 were based on a combination of search strategies from Betty et al. (2015), Sutton et al. (2020) and Kilsdonk et al. (2011) examining the literature on clinical decision support systems.
20. Line 16 "decision support systems, clinical"[MeSH Terms] was added as a MeSH term. It was introduced as a MeSH term in 1998 and described as computer-based information systems used to integrate clinical and patient information and provide support for decision-making in patient care.
21. Line 17 "Artificial Intelligence"[MeSH Terms] was added as a MeSH term. It was introduced as a MeSH term in 1986 and described as the theory and development computer systems which perform tasks that normally require human intelligence.
22. Line 18 "clinical decision support system*"[Title/Abstract] was added to capture anything missed by the MeSH headings and to capture “clinical decision support systems” or other similar ending terms
23. Line 19 and 20 "computerised decision support system*"[Title/Abstract] and "computerized decision support system*"[Title/Abstract] were used specifically to capture language variation. A wildcard wasn’t used on advice from medical librarian and for ease of use when transferring the search to other databases.
24. Line 19"computerised decision support system*"[Title/Abstract] was added to capture “computerised decision support systems” and other similar ending terms.
25. Line 20 "computerized decision support system*"[Title/Abstract] was added to capture “computerized decision support systems” and other similar ending terms.
26. Line 21 "decision support*"[Title/Abstract] was used in Betty et al. (2015) search strategy and it was added to capture “decision supports” and other similar ending terms.
27. Line 22"cdss"[Title/Abstract] was added to capture any articles using abbreviations of “clinical decision support system” or “computerised decision support system”. Phrase and title abstract searching were used to keep the search relevant.
28. Line 23 "Artificial Intelligence"[Title/Abstract] was added to capture any articles missed by the MeSH headings.
29. Line 24 “"AI"[Title/Abstract] was added to capture any articles using the abbreviation of “artificial intelligence. Phrase and title abstract searching were used to keep the search relevant.
30. Line 25 "reminder system*"[Title/Abstract] was added to capture examples of common CDSS and to capture “reminder systems” or other similar ending terms.
31. Line 26 "electronic feedback"[Title/Abstract] was specifically added to capture examples of common CDSS. PubMed included many irrelevant results and irrelevant endings with “electronic feedback *”.
32. Line 27 "dashboard*"[Title/Abstract]) was added to capture examples of common CDSS and to capture “dashboards” or other similar ending terms.
33. Line 29 ("Hospitals"[MeSH Terms] was added to capture the hospital setting. The term describes institutions with an organized medical staff which provide medical care to patients.
34. Line 30 ("hospital*"[Title/Abstract] was added to capture any terms missed from the MeSH heading and to capture “hospitals”, “hospitalisations” and other similar ending terms.
35. Line 31 and Line 32 "medical centre*"[Title/Abstract] and "medical center*"[Title/Abstract])) were used specifically to capture language variation. A wildcard wasn’t used on advice from medical librarian and for ease of use when transferring the search to other databases. A MeSH term for medical centre could not be found with the closest MeSH term being “academic medical centre” which may bias the results.
36. No search restrictions (i.e. English only, human not animal) applied.

# Pubmed

**Platform** Embase®

**Databases** EMBASE via Elsevier

**Search date coverage** No date limit

**Search strategy**

1. **'health care planning'**/exp
2. 'health care planning'
3. **'implementation science'**/exp
4. 'implementation science'
5. 'health care planning’: ab,ti
6. 'implementation science’: ab,ti
7. 'implement*’: ab,ti
8. ‘adopt*’: ab,ti
9. OR/1-8
10. 'framework*’: ti,ab
11. 'model*’: ti,ab
12. 'theor*’: ti,ab
13. 'mapping’: ti,ab
14. 'program*’: ti,ab
15. 'tool*’: ti,ab
16. 'assessment*’: ti,ab
17. OR/10-16
18. **'clinical decision support system'**/exp
19. **'artificial intelligence'**/exp
20. 'clinical decision support system*’: ti,ab
21. 'computerised decision support system*’: ti,ab
22. 'computerized decision support system*’: ti,ab
23. 'decision support*’: ti,ab
24. 'cdss’: ti,ab
25. 'artificial intelligence’: ti,ab
26. 'ai’: ti,ab
27. 'reminder system*’: ti,ab
28. 'electronic feedback’: ti,ab
29. 'dashboard*’: ti,ab
30. OR/18-29
31. **'hospital'/exp**
32. **'medical centers'/exp**
33. **'medical center'/exp**
34. 'hospital*’: ti,ab
35. 'ward*’: ti,ab
36. 'medical centre*’: ti,ab
37. 'medical center*’: ti,ab
38. OR/31-37
39. 9 AND 17 AND 30 AND 38
40. medline]/lim
41. NOT/40

**­Notes**

1. Line 1 **'health care planning'**/exp was included as an emtree term to be consistent with Pubmed search. The term was added in 1987 and defined along the lines of community health planning; health and welfare planning; health plan implementation; health planning; health planning guidelines; health planning organisations, health planning support etc.
2. Line 2 'health care planning' was added to capture any terms missed by the emtree heading and to be consistent with the Pubmed search.
3. Line 3 **'implementation science'**/exp was added to an emtree term. The term was added in 2019 and describes implementation research and implementation scientific research.
4. Line 4 'implementation science' was added as a phrase search to capture any missing terms from emtree headings and title/abstract search. Around 7184 results were found compared to around 4093 results from emtree headings and title/abstract search of line 3 and 5.
5. Line 8 ‘adopt*’: ti,ab was added to capture terms such as “adopt” “adoption” or “adopts” and others similar endings as based on expert peer reviewer comments that the field of HIT evaluation / medical informatics use terms other than implementation to describe the implementation process
6. Line 18 **'clinical decision support system'**/exp was added as an exploded emtree heading. The term was added in 2016 and described CDS system (clinical decision support system); clinical decision support (CDS) system; clinical decision support systems; clinician decision support system and decision support systems, clinical.
7. Line 19 **'artificial intelligence'**/exp was added as an exploded emtree term. The term was added in 1974 and describes a branch of computer science in which machines are made to perform functions similar to the intellectual functions of the human mind, such as learning and reasoning.
8. Lines 20-23 were included to be consistent to PubMed search and to capture articles missed by the emtree heading.
9. Lines 21 and 22 'computerised decision support system*’: ti,ab and 'computerized decision support system*’: ti,ab were added to be consistent to PubMed search and to account for language variation.
10. Line 23'decision support*’: ti,ab although there is an emtree term for ‘decision support’ this was not added to the search as **'clinical decision support system'** fell under this emtree term and was more specific to the search being conducted.
11. Line 24-line 29was included to be consistent to pubmed search and to capture articles missed by the emtree heading.
12. Line 31 **'hospital'/exp** was added as an exploded emtree heading. The term was added in 1974 and describes many different hospitals settings such as emergency hospital; environment, hospital; hospital data; hospital environment; hospital establishment; regional hospital; state hospital; voluntary hospital etc.
13. Line 32 **'medical centers'/exp** was a candidate emtree term that was included to capture language variation and any missing/specific articles.
14. Line 33 **'medical center'/exp** was a candidate emtree term that was included to capture language variation and any missing/specific articles.
15. Line 34-37 included to stay consistent with Pubmed searches and in particular lines 35 and 36'medical centre*’: ti,ab and 'medical center*’: ti,ab were added to account for language variation.
16. Line 39 medline]/lim was added on recommendation from medical librarian with scoping review experience to exclude Medline results. This removes potential duplicates from the PubMed search and allows for potential new articles to be captured.
17. No other search restrictions (i.e. English only, human not animal) applied.

**Platform** EBSCO

**Databases** Cumulative Index to Nursing and Allied Health Literature (CINAHL)

**Search date coverage** No date limit

**Search strategy**

1. MH "Health Facility Planning+"
2. MH "Implementation Science"
3. TI “implement*”
4. AB “implement*”
5. TI “Health Facility planning”
6. AB “Health Facility planning”
7. TI “Implementation Science”
8. AB “Implementation Science”
9. TI “Adopt*”
10. AB “Adopt*”
11. OR/1-10
12. TI “framework*”
13. AB “framework*”
14. TI “model*”
15. AB “model*”
16. TI “theor*”
17. AB “theor*”
18. TI “Mapping”
19. AB “Mapping”
20. TI “program*”
21. AB “program*”
22. TI “tool*”
23. AB “tool*”
24. TI “assess*”
25. AB “assess*”
26. OR/12-25
27. MH "Decision Support Systems, Clinical"
28. MH "Artificial Intelligence+"
29. TI "clinical decision support system*”
30. AB "clinical decision support system*"
31. TI "computerised decision support system*"
32. AB "computerised decision support system*"
33. TI "computerized decision support system*"
34. AB "computerized decision support system*"
35. TI "decision support*"
36. AB "decision support*"
37. TI “cdss”
38. AB “cdss”
39. TI "Artificial Intelligence"
40. AB "Artificial Intelligence"
41. TI “AI”
42. AB “AI”
43. TI "reminder system*"
44. AB "reminder system*"
45. TI "electronic feedback"
46. AB "electronic feedback"
47. TI “dashboard*”
48. AB “dashboard*”
49. OR/ 27-28
50. MH "Hospitals+"
51. TI “hospital*”
52. AB “hospital*”
53. TI “ward*”
54. AB “ward*”
55. TI "medical centre*"
56. AB "medical centre*"
57. TI "medical center*"
58. AB "medical center*"
59. OR/50-58
60. 11 AND 26 AND 49 AND 59

**Limiters** - Full Text

**Expanders** - Apply equivalent subjects

**Search modes** - Boolean/Phrase

**­Notes**

1. Line 1 MH "Health Facility Planning+" was added as an exploded major heading to be consistent with the terms ‘health plan implementation’ and ‘health care planning’ in the PubMed and Embase searches. It is described as area-wide planning for healthcare institutions on the basis of projected consumer needs and demand and includes setting specific hospital planning which describes strategy of building of hospitals or a specific hospital unit on the basis of projected consumer needs and demand.
2. Line 2 MH "Implementation Science" was added as a major heading to be consistent with Pubmed searches and the term describes the study of promoting evidence-based practices and policies and their adoption.
3. Line 3-4 TI “implement*” and AB “implement*” was added to capture missing terms from major heading searches and to be consistent with PubMed search strategy.
4. Lines 5-6 TI “Health Facility planning” and AB “Health Facility planning” was added to capture missing terms from the major heading search.
5. Lines 7-8 TI “Implementation Science” and AB “Implementation Science” was added to capture missing terms from major heading searches and to be consistent with PubMed search strategy.
6. Line 9-10 TI “Adopt*” AB “Adopt*” was added to capture terms such as “adopt” “adoption” or “adopts” and others similar endings as based on expert peer reviewer comments that the field of HIT evaluation / medical informatics use terms other than implementation to describe the implementation process
7. Lines 12-25 was added to be consistent with the PubMed search strategies.
8. Line 27 MH "Decision Support Systems, Clinical" was added as a major heading to be consistent with PubMed and Embase terms and describes systems that integrate clinical data and patient data to support the decisions made by the clinicians. These systems contain numerous databases of information such as drug interactions and contraindications, treatment costs, clinical practice guidelines, and critical paths.
9. Line 28 MH "Artificial Intelligence+" was added as an exploded major heading to be consistent with the PubMed search. It describes the capacity of a device to perform functions normally associated with human intelligence, such as reasoning, learning and self-improvement and includes major headings for the uses of AI such as expert systems, knowbots, knowledge bases, machine learning, natural language processing, neural networks (computer), robotics and blockchain.
10. Line 39-36 was added to stay consistent to PubMed search and capture any missing terms from the major heading search for line 25 MH "Decision Support Systems, Clinical".
11. Line 35-36 TI "decision support*" and AB "decision support*" was specifically included to stay consistent to PubMed searches and as there was no major heading for “decision support” with the closest major heading being line 25 MH "Decision Support Systems, Clinical".
12. Line 39-42 was added to be consistent to the PubMed search and to capture any missing terms from the major heading search for line 26 MH "Artificial Intelligence+"
13. Line 31-34 were added to be consistent to PubMed searches and to capture any language variation.
14. Line 43-48 was added to be consistent to PubMed search and to capture articles missed by the major heading searches.
15. Line 50 MH "Hospitals+" was added as an exploded major heading. The term describes an institution that provides care and treatment for the sick and injured. The term also includes other major headings such as hospitals, community; hospitals, private; hospitals, public; hospitals, rural; hospitals, special; hospitals; urban; magnet hospitals and housing for the elderly.
16. Line 51-54 were added to be consistent to PubMed searches and to capture any missing terms or articles from line 48 major heading search MH "Hospitals+"
17. Line 55-58 were added to be consistent to PubMed searches and to account for language variation.
18. Line 55-58 was added as like PubMed the closest major heading was ‘academic medical centres’ which may have biased the results.
19. No other search restrictions (i.e. English only, human not animal) applied.

**Platform** Elsevier

**Databases** Scopus

**Search date coverage** No date limit

**Search strategy**

1. INDEXTERMS (“Health Plan Implementation”)
2. INDEXTERMS (“Implementation Science”)
3. TITLE-ABS (“implement*”)
4. TITLE-ABS (“Health Plan Implementation”)
5. TITLE-ABS (“Implementation Science “)
6. TITLE-ABS(“Adopt*”)
7. OR/1-6
8. TITLE-ABS (“framework*”)
9. TITLE-ABS (“model*”)
10. TITLE-ABS (“theor*”)
11. TITLE-ABS (“Mapping”)
12. TITLE-ABS (“program*”)
13. TITLE-ABS (“tool*”)
14. TITLE-ABS (“assess*”)
15. OR/8-14
16. INDEXTERMS (“decision support systems, clinical”)
17. INDEXTERMS (“Artificial Intelligence”)
18. TITLE-ABS (“clinical decision support system*”)
19. TITLE-ABS (“computerised decision support system*”)
20. TITLE-ABS (“computerized decision support system*”)
21. TITLE-ABS (“decision support*”)
22. TITLE-ABS (“cdss”)
23. TITLE-ABS (“Artificial Intelligence”)
24. TITLE-ABS (“AI”)
25. TITLE-ABS (“reminder system*”)
26. TITLE-ABS (“electronic feedback”)
27. TITLE-ABS (“dashboard*”)
28. OR/16-27
29. INDEXTERMS (“Hospital”)
30. TITLE-ABS (“hospital*”)
31. TITLE-ABS (“ward*”)
32. TITLE-ABS (“medical centre*”)
33. TITLE-ABS (“medical center*”)
34. OR/29-33
35. 7AND 15 AND 28 AND 34

**­Notes**

1. Line 1 INDEXTERMS (“Health Plan Implementation”) was added to be consistent with the PubMed search
2. Line 2 INDEXTERMS (“Implementation Science”) was added to be consistent with the PubMed search
3. Lines 3-5 was added to be consistent with the PubMed search and to capture any missing data from the Index terms searches in line 1 and line 2.
4. Line 6 TITLE-ABS(“Adopt*”) was added to capture terms such as “adopt” “adoption” or “adopts” and others similar endings as based on expert peer reviewer comments that the field of HIT evaluation / medical informatics use terms other than implementation to describe the implementation process
5. Line 8-14 was added to be consistent with the PubMed search
6. Line 16 and line 17 INDEXTERMS (“decision support systems, clinical”) and INDEXTERMS (“Artificial Intelligence”) was added to be consistent with the PubMed search
7. Line 18-27 was added to be consistent with the PubMed search and to capture any missing data from the Index terms searches in line 15 and line 16.
8. Line 19 and 20 TITLE-ABS (“computerised decision support system*”) and TITLE-ABS (“computerized decision support system*”) was added to account for language variation
9. Line 29 INDEXTERMS (“Hospital”) was added to be consistent with the PubMed search
10. Line 31-32 was added to be consistent with the PubMed search and to capture any missing data from the Index terms searches in line 28.
11. Line 32 and 33 TITLE-ABS (“medical centre*”) and TITLE-ABS (“medical center*”) was added to account for language variation
12. No other search restrictions (i.e. English only, human not animal) applied.

**Databases** Web of Science

**Search date coverage** No date limit

**Search strategy**

1. TS= (“Health Plan Implementation”)
2. TS= (“Implementation Science”)
3. TI= (“Health Plan Implementation”)
4. AB= “Health Plan Implementation”
5. TI= “Implementation Science”
6. AB= “Implementation Science”
7. TI= “implement*”
8. AB= “implement*”
9. TI= “adopt*”
10. AB= “adopt*”
11. OR/1-10
12. TI= “framework*”
13. AB= “framework*”
14. TI= “model*”
15. AB= “model*”
16. TI= “theor*”
17. AB= “theor*”
18. TI= “Mapping”
19. AB= “Mapping”
20. TI= “program*”
21. AB= “program*”
22. TI= “tool*”
23. AB= “tool*”
24. TI= “assess*”
25. AB= “assess*”
26. OR/12-25
27. TS= “clinical decision support system*”
28. TS= “Artificial Intelligence”
29. TI= “clinical decision support system*”
30. AB= “clinical decision support system*”
31. TI= “computerised decision support system*”
32. AB= “computerised decision support system*”
33. TI= “computerized decision support system*”
34. AB= “computerized decision support system*”
35. TI= “decision support*”
36. AB= “decision support*”
37. TI= “cdss”
38. AB= “cdss”
39. TI= “Artificial Intelligence”
40. AB= “Artificial Intelligence”
41. TI= “AI”
42. AB= “AI”
43. TI= “reminder system*”
44. AB= “reminder system*”
45. TI= “electronic feedback”
46. AB= “electronic feedback”
47. TI= “dashboard*”
48. AB= “dashboard*”
49. OR/27-48
50. TS= “Hospital”
51. TI= “hospital*”
52. AB= “hospital*”
53. TI= “ward*”
54. AB= “ward*”
55. TI= “medical centre*”
56. AB= “medical centre*”
57. TI= “medical center*”
58. AB= “medical center*”
59. OR/50-58
60. 11 AND 16 AND 29 AND 35

**Notes**

1. Although, initial searches were conducted in IEEE-explore and ACM two computer science and technology databases the searches were difficult to conduct or navigate with wild card restrictions/key word restrictions and too many results. Thus, Web of science was to capture technology and computer science-based articles on recommendation from a medical librarian with experience in conducting scoping reviews.
2. Line 1 and line 2 TS= ("Health Plan Implementation") and TS= ("Implementation Science") were topic sentences added to be consistent with PubMed and other searches.
3. Line 4-8 was added to be consistent with the PubMed search and to capture any missing data from the topic searches in line 1 and line 2.
4. Line 9-10 TI= “adopt*” and AB= “adopt*”
5. was added to capture terms such as “adopt” “adoption” or “adopts” and others similar endings as based on expert peer reviewer comments that the field of HIT evaluation / medical informatics use terms other than implementation to describe the implementation process
6. Line 12-25 added to be consistent with the PubMed search
7. Line 27 and 28 TS=” clinical decision support system*” and TS=” Artificial Intelligence” topic searches was added to be consistent with PubMed and other searches examining CDSS and artificial intelligence
8. Line 29-48 was added to be consistent with the PubMed search and to capture any missing data from the topic searches in line 27 and line 28.
9. Lines 31-34 was added to capture language variation
10. Line 50 TS=” Hospital” topic search was added to be consistent with the PubMed and other search strategies
11. Line 51-58 was added to be consistent with the PubMed search and to capture any missing data from the topic searches in line 50
12. Line 55-58 was added to capture language variation
13. No other search restrictions (i.e. English only, human not animal) applied.
