# Supplementary material for: Using Theories, Models, and Frameworks to Inform Implementation Cycles of Computerized Clinical Decision Support Systems in Tertiary Health Care Settings: Scoping Review
Source: J Med Internet Res. 2023 Oct 18;25:e45163. doi: 10.2196/45163 (PMC10620641; doi:10.2196/45163)
Supplement: Multimedia Appendix 3 [file jmir_v25i1e45163_app3.doc]

Multimedia Appendix 3

Definitions and elaborations document

**Key words and definitions:**

**Theory**

A theory is often a set of analytical principals or statements designed to explain our observations or understandings of the world.

Theories explain how and why specific relationships lead to specific phenomena.

Therefore, in the field of implementation science, theories imply predictions and attempt to explain causal mechanisms for implementation

Examples of theories: Theory of Diffusion, social cognitive theories, theories concerning cognitive processes and decision making, social networks theories, social capital theories, communities of practice, professional theories, organizational theories. Implementation Climate, Absorptive Capacity, Organizational Readiness, COM-B, Normalization Process Theory [30].

**Model**

Do not specify mechanisms for change and act as checklists for implementation factors

Often include linear or iterative steps, phases or stages describing the translation of research into practice

Describes the simplification of a phenomena and in implementation science and is often used to guide the process of translating research into practice

Examples of models: CIHR Model of Knowledge Translation, the K2A Framework, the Stetler Model, the ACE Star Model of Knowledge Transformation, the Knowledge-to-Action Model, the Iowa Model, the Ottawa Model; the Quality Implementation Framework [30].

**Framework**

Do not specify mechanisms for change and act as checklists for implementation factors

Denotes the overview or outline of various descriptive categories e.g., concepts, constructs, or variables and presupposes the relationships between categories lead to a phenomenon

Implementation Science often describes implementation factors found to influence implementation outcomes e.g., examining the domain of the policy context in the wider system for technology adaption

Examples of frameworks: PARIHS, Active Implementation Frameworks, Understanding-User-Context Framework, Conceptual Model, Theoretical Domains Framework, RE-AIM; PRECEDE-PROCEED; framework by Proctor et al. [30]

**Implementation**

Being implemented prospectively

Exclude retrospective evaluations of CDSS using theory, model, or framework. Retrospective evaluations are often not real time examinations of planned implementation strategies in practice, but theory, model or framework facilitated examinations of effective versus ineffective implementation or intervention adoption processes already conducted.

However, some evaluations can inform data collection and implementation activities and are planned well before implementation of the CDSS.

Exclude articles purely about knowledge synthesis which is the rigorous and systematic generation of evidence-based products (patents, materials, tools, programs, and guidelines) for application in policy and practice or the development of a CDSS tool.

Include articles on implementation which refers to the rigorous application of new knowledge into policy and practice in a theory informed and reflective way.

**Clinical decision support system**

A computerised clinical decision support system would be software designed to be a direct aid to clinical decision making at the point of care [1].

Any studies examining patient decision making as opposed to clinical decision making may be excluded. Factors influencing uptake and adoption of CDSS that reflected how digital health is utilised within health systems and research shows the decisions are made from health system perspective and not primarily the patient perspective since CDSS form part of a clinical process without patient involvement [54].

CDSS can include web-applications, computerised provider order entry (CPOE) systems, desktop, smart phone, tablet, and devices such as biometric monitoring or wearable health technology which can be linked to electronic health record databases [1].

**Tertiary healthcare settings**

Include university hospital settings

In other countries such as America sometimes hospitals are referred to as medical centres

Inpatient and outpatient care

The setting hospital usually falls under tertiary care, or the terms are utilised interchangeably [55-57]

| Table S1. Inclusion and exclusion criteria | |
| --- | --- |
| Eligibility criteria | Criteria |
| **Inclusion**  **Criteria** | Examined implementation of a CDSS^b^ into clinical or routine practice |
|  | CDSS designed for use at the point of care |
|  | Prospective implementation of CDSS |
|  | Conducted in a hospital or tertiary healthcare setting |
|  | Reported using an implementation theory, model or framework in relation to the implementation of a CDSS ^a^ |
| **Exclusion**  **Criteria** | Conducted solely in a primary care or community setting such as general practice, allied health care clinics |
|  | Developed a theory, model or framework but did not apply it to a CDSS implementation |
|  | Described the development of a CDSS |
|  | Retrospectively evaluated a CDSS |
|  | Studied patient decision making as opposed to clinical decision making |
| ^a^ Definitions/elaboration of terms used to identify theories, models or frameworks according to Nilsen’s Taxonomy [30] have been provided in Multimedia appendix 2.  ^b^ Computerized Clinical Decision Support System | |
