# Supplementary material for: Using Theories, Models, and Frameworks to Inform Implementation Cycles of Computerized Clinical Decision Support Systems in Tertiary Health Care Settings: Scoping Review
Source: J Med Internet Res. 2023 Oct 18;25:e45163. doi: 10.2196/45163 (PMC10620641; doi:10.2196/45163)
Supplement: Multimedia Appendix 4 [file jmir_v25i1e45163_app4.docx]

**Identification of studies via databases**

Records removed *before screening*:

Duplicate records removed

(n = 2584)

Records identified from:

Databases (N =5995)

PubMed (n=1369)

CINAHL (n= 635)

Embase (n= 1145)

Scopus (n= 1892)

Web of Science (n= 954)

**Identification**

Records screened

(n =3411)

Records excluded

(n = 3228)

Reports sought for retrieval

(n =183)

Reports not retrieved

(n = 1)

**Screening**

Reports excluded (n=139):

Abstracts (n=34)

- Duplicate (n=4)
- Does not define TMF (n=11)
- Not considered computerised CDSS (n=10)
- TMF not used for implementation (evaluation) (n=3)
- Development of CDSS (n=5)
- TMF developed but not applied to CDSS Implementation (n=1)

Full Text (n=105)

- Duplicate (n=3)
- Does not define TMF (n=10)
- Not considered computerised CDSS (n=14)
- TMF not used for implementation (evaluation) (n=25)
- Development of CDSS (n=36)
- Not a hospital/tertiary care setting (n=5)
- TMF developed but not applied to CDSS Implementation (n=11)
- Not published in a peer reviewed publication (n=1)

Reports assessed for eligibility

(n =183)

Abstracts (n=55)

Full text (n=128)

**Studies included in review (n=44)**

**Full Text (n=23)**

**Abstracts (n=21)**

**Included**
