# Supplementary material for: Using Theories, Models, and Frameworks to Inform Implementation Cycles of Computerized Clinical Decision Support Systems in Tertiary Health Care Settings: Scoping Review
Source: J Med Internet Res. 2023 Oct 18;25:e45163. doi: 10.2196/45163 (PMC10620641; doi:10.2196/45163)
Supplement: Multimedia Appendix 5 [file jmir_v25i1e45163_app5.docx]

Multimedia Appendix 5

Abstracts included

| Table 3. Characteristics of included abstracts (n=39) | | | | | | | |
| --- | --- | --- | --- | --- | --- | --- | --- |
| Nilsen’s taxonomy | First author reference | Country **^a^** | Data type | Study design | Clinical context | CDSS type | Theory, model or framework |
| **Model** | Arenal López, Najera [107] | Not stated | Not stated | Not stated | Prescription | Graph displayed on computer at ICU bedside | Lopez' et al's process model (three steps) |
|  | Azul, Kreppel [108] | United States | Mixed | Retrospective cohort study | Venous thromboembolism screening | Electronic reminders | Plan, Do, Study, Act Cycle |
|  | Bosma, Bechtold [109] | United States | Quant **^b^** | Not stated | Hospital acquired infections | Clinical Dashboard | Plan, Do, Study, Act Cycle |
|  | Buenger and Webber [110] | Not stated | Quant | Pre-post | Vaccination | Alerts in EHR | LEAN Methodology |
|  | Dalal, Fuller [111] | United States | Mixed | Exploratory analysis | Hospital acquired infections | Patient safety Dashboard | Conceptual Integration Framework |
|  | Disabato, Levisohn [112] | United States | Mixed | Pre-post | Epilepsy care | Alert & smart form in electronic health record (EHR) | Plan, Study, Do, Act Cycle |
|  | Elshenawy, O'Connor [113] | United States | Quant | Pre-post | MRI sedation guidelines | Clinical dashboard | Plan, Do, Study, Act Cycle |
|  | Federico, Redmond [114] | United States | Quant | Pre-post | Asthma management | EHR & alert | Plan, Study, Do, Act Cycle |
|  | Hassanain, Zamakhshary [115] | Saudi Arabia | Quant | Pre-post | Operational room patient management | Visual dashboard & electronic list | LEAN Methodology |
|  | Hemmat, Wang [116] | United States | Quant | Pre-post | Opioid disorder prescribing | her  Decision support | Plan, Do, Study, Act Cycle |
|  | Hsu, Wang [117] | Taiwan | Mixed | Pre-post | Prescription | CDSS in EHR | Hsu's process model (five steps) |
|  | Hung, Yang [118] | Taiwan (Republic of China) | Quant | Retrospective cohort study | Fracture prevention | Artificial intelligence | Five 'I' Model |
|  | Keller, Thompson [119] | Not stated | Quant | Pre-post | Patient management | Form within HER | Plan, Do Study Act cycle |
|  | Ketcham, Fredella [120] | United States | Quant | Quasi-experimental cohort study | Prescription | EHR tracking board. | Six-sigma |
|  | Khatri, Carl [121] | United States | Mixed | Pre-post | Asthma Screening | Clinical guideline algorithm in HER | Plan Do Study Act cycle |
|  | Levasseur Sanchez, Kanna [122] | United States | Quant | Pre-post | Asthma management | Electronic reminder | Plan, study, Do, Act Cycle |
|  | Mazloom, Steffen [123] | United States | Mixed | Case Study | Blood stream infections | Electronic reminder | Plan, Do, Study, Act Cycle |
|  | Miller, Chara [124] | Not stated | Quant | Pre-post | Paediatric screening | CDSS in electronic medical record | Plan, Do, Study, Act Cycle |
|  | Norris, Parekh [125] | United States | Quant | Pre-post | Cardiac monitoring | Electronic reminder | Plan, Do, Study, Act Cycle |
|  | Obringer and Bartlett [126] | United States | Mixed | Pre-post | Influenza vaccination | Alert | Plan, Do, Study, Act Cycle |
|  | Rungvivatjarus, Vuong [127] | Not stated | Quant | Pre-post | Patient monitoring | CDSS within HER | Plan, Do, Study, Act Cycle |
|  | Schwartzberg and Selitzky [128] | Israel | Mixed | Pre-post | Pharmacy | Online calculator | ‘Deming’ quality circle, Plan, Do, Check & Do |
|  | Sharif, Natarajan [129] | Not stated | Quant | Pre-post | Medical imaging | Electronic alert | IHI "model for improvement" Plan Do Study Act Cycle |
|  | Karim [130] | Pakistan | Quant | Pre-post | Blood component ordering | Computer physician order entry system (CPOE) | Plan, Do, Study, Act Cycle |
|  | Simões, Maia [131] | Portugal | Not Specified | Case Series | Anti-biotic prescription | HAI- tool - Information System | Design Science Research Methodology (DSRM) |
|  | Singh, Morrissey [132] | Not stated | Quant | Pre-post | Prescription | Alert within HER | Plan Do Study Act cycle |
|  | Yeh, Dworsky [133] | United States | Quant | Pre-post | Nutrition | Screening tool and alert within EHR | Plan Do Study Act cycle |
| **Determinant framework** | Holdsworth, Kling [134] | Not stated | Mixed | Stepped Wedge Trial | Clinical Deterioration | AI prediction monitoring | Systems Engineering Initiative for Patient Safety (SEIPS) 2.0 model |
|  | Hunt, Kirkham [135] | Canada | Mixed | Pre-post | Concussion treatment | Integrated software platform | International Classification of Functioning Disability and Health framework |
|  | Jordan, Hauser [136] | United States | Qual | Exploratory qualitative study | Patient management | Artificial intelligence | Campinha-Bacote's theory of cultural competence |
|  | Karim, Fegeler [137] | Not stated | Mixed | Pre-post | Medical imaging | Web-based electronic form | Technology Acceptance Model |
| **Determinant Frameworks** | Snader, Soh [138] | Not stated | Qual | Exploratory qualitative study | Not stated | CDSS alerts | Theoretical Domains Framework (TDF) & Technology Acceptance Framework (TAF) |
| **Evaluation framework** | Gross, Thompson [139] | United States | Quant | Retrospective cohort study | Albumin ordering | Order set & decision support | Precede- Proceed |
| **Theory** | Macheel, Reicks [140] | Not stated | Quant | Pre-post | Rib fracture treatment | Order set | Unified theory of acceptance & use of technology (UTAUT) |
| **Model and theory** | Steinmo, Voegele [141] | Not stated | Mixed | Pre-post | Patient monitoring | Electronic Dashboard | Plan Do Study Act cycle & Capability Opportunity Motivation Model of Behaviour (COM-B) |
| **Insufficient evidence** | Moreno-Franco, Grek [142] | United States | Quant | Quasi-experimental time-series | Sepsis treatment | Decision Support tools & her | Insufficient evidence to classify |
|  | DeNardo, Renaud [143] | United States | Mixed | Pre-post | Pediatic oncology | CPOE | DeNardo et al. framework |
|  | Kibert, Vinluan [144] | United States | Not specified | Case study | Anticoagulation | Population  Management Dashboard | Change in Practice Model |
|  | Thompson, Kell [145] | United States | Mixed | Pre-post | Heart failure | Clinical Dashboard | LEAN methodology |
